# The Slowdown of Growth Rate Controls the Single-Cell Distribution of Biofilm Matrix Production via an SinI-SinR-SlrR Network

**DOI:** 10.1128/msystems.00622-22

**Published:** 2023-02-14

**Authors:** Zhuo Chen, Brenda Zarazúa-Osorio, Priyanka Srivastava, Masaya Fujita, Oleg A. Igoshin

**Affiliations:** a Systems, Synthetic and Physical Biology Program, Rice University, Houston, Texas, USA; b Department of Biology and Biochemistry, University of Houston, Houston, Texas, USA; c Department of Bioengineering, Rice University, Houston, Texas, USA; d Department of Chemistry, Rice University, Houston, Texas, USA; e Department of Biosciences, Rice University, Houston, Texas, USA; f Center for Theoretical Biological Physics, Rice University, Houston, Texas, USA; Technical University of Denmark

**Keywords:** biofilms, biosystems, gene expression, stochasticity

## Abstract

In Bacillus subtilis, master regulator Spo0A controls several cell-differentiation pathways. Under moderate starvation, phosphorylated Spo0A (Spo0A~P) induces biofilm formation by indirectly activating genes controlling matrix production in a subpopulation of cells via an SinI-SinR-SlrR network. Under severe starvation, Spo0A~P induces sporulation by directly and indirectly regulating sporulation gene expression. However, what determines the heterogeneity of individual cell fates is not fully understood. In particular, it is still unclear why, despite being controlled by a single master regulator, biofilm matrix production and sporulation seem mutually exclusive on a single-cell level. In this work, with mathematical modeling, we showed that the fluctuations in the growth rate and the intrinsic noise amplified by the bistability in the SinI-SinR-SlrR network could explain the single-cell distribution of matrix production. Moreover, we predicted an incoherent feed-forward loop; the decrease in the cellular growth rate first activates matrix production by increasing in Spo0A phosphorylation level but then represses it via changing the relative concentrations of SinR and SlrR. Experimental data provide evidence to support model predictions. In particular, we demonstrate how the degree to which matrix production and sporulation appear mutually exclusive is affected by genetic perturbations.

**IMPORTANCE** The mechanisms of cell-fate decisions are fundamental to our understanding of multicellular organisms and bacterial communities. However, even for the best-studied model systems we still lack a complete picture of how phenotypic heterogeneity of genetically identical cells is controlled. Here, using B. subtilis as a model system, we employ a combination of mathematical modeling and experiments to explain the population-level dynamics and single-cell level heterogeneity of matrix gene expression. The results demonstrate how the two cell fates, biofilm matrix production and sporulation, can appear mutually exclusive without explicitly inhibiting one another. Such a mechanism could be used in a wide range of other biological systems.

## INTRODUCTION

To adapt to various environmental conditions, bacterial cells can differentiate into different cell types (1). Bacillus subtilis is one of the best-understood model systems for studying bacterial cell differentiation. Upon starvation, a subpopulation of B. subtilis cells can differentiate into matrix producers, which secrete extracellular biofilm matrix (2, 3). As a result, given the right environmental conditions, cells encase themselves in the extracellular matrix and thereby form a biofilm (4). At later stages of starvation, B. subtilis cells can further activate another cell differentiation pathway and transition into spores (5). Notably, in biofilms, different cell types coexist, forming a highly heterogeneous community (6, 7).

Both the matrix production and sporulation are activated by the same transcriptional master regulator, Spo0A. Upon starvation, Spo0A is phosphorylated to become an active form as a transcription factor (Spo0A~P) through a multicomponent phosphorelay composed of five kinases on the top and two intermediate phosphotransferases (8, 9). Among five kinases, KinA and KinC play major roles to control sporulation and biofilm formation, respectively (10–12). The cellular concentration of Spo0A~P gradually increases in a pulsatile manner over the course of starvation, leading to up-/downregulation of genes and operons having binding sites (named 0A-box) for the phosphoproteins (10, 13–15). However, many of the 0A boxes deviate from the consensus sequence (5′-TGTCGAA-3′) (13). Thus, the binding affinity of Spo0A~P to the 0A-box changes with the variation of the consensus sequence (10). The amplitude of Spo0A~P increases as starving cells accumulate KinA due to a decrease in growth rate (11, 15). At early times of starvation, the relatively low concentrations of Spo0A~P preferentially bind to the high-affinity 0A-boxes in the genes and operons involved in biofilm matrix production. When starvation persists, the high dose of Spo0A~P occupies the weak-affinity sites in the genes and operons involved in sporulation (10, 16).

**FIG 1:**
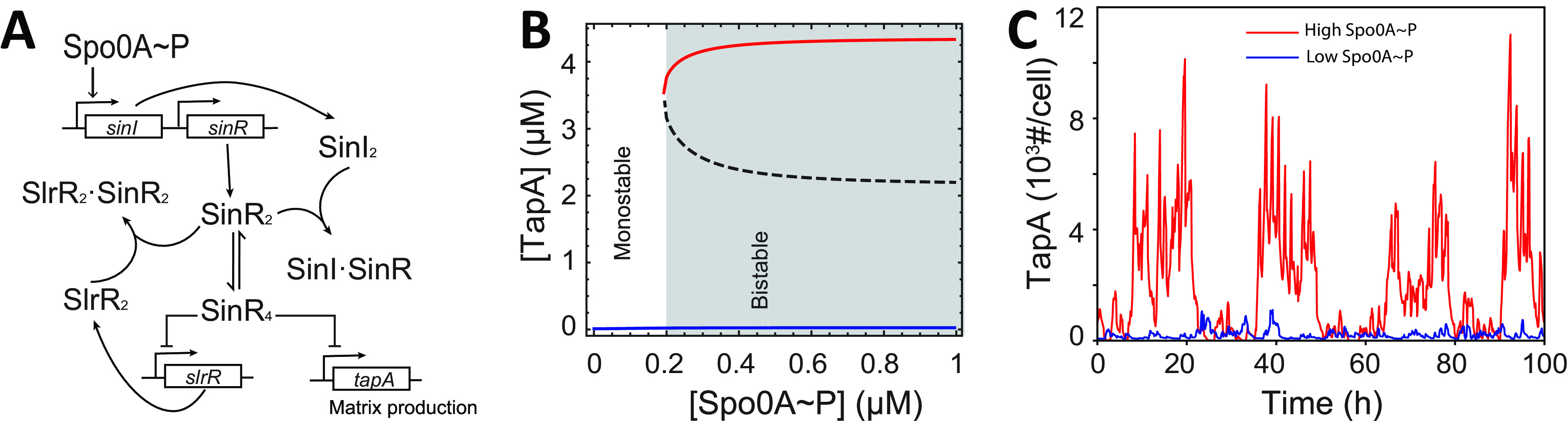
Bistable expression of *tapA* is controlled by the Spo0A~P level via the SinI-SinR-SlrR network. (A) The schematic of the SinI-SinR-SlrR network used in our model (see Materials and Methods for details). (B) Deterministic model of the network predicts the existence of two steady states, namely, high (red) and low (blue), of the TapA steady-state concentration. At low Spo0A~P concentrations (unshaded region), only a low steady state exists (monostability). At high Spo0A~P concentrations (shaded in gray), two stable steady states coexist in the bistable region. Unstable steady state separating the two is show by dashed line. (C) Stochastic simulation of *tapA* expression as a function of time was performed using the fixed Spo0A~P concentrations at low (0.05 μM, blue line, value from the monostable region in B) or at high values (1 μM red line, value from the bistable region in B). The *x* and *y* axis indicate time (h), and TapA reporter levels are shown in the number of molecule per cell(#/cell).

While genes involved in sporulation are directly controlled by Spo0A~P, genes involved in biofilm matrix production are controlled via an additional SinI-SinR-SlrR network, which is also under the control of Spo0A~P, leading to the expression of a set of genes and operons, including the *tapA* (formerly named *yqxM*)-*sipW*-*tasA* operon and the eps operon (Fig. 1A) (17, 18). At the top of the regulatory network, Spo0A~P activates the expression of *sinI* (19). Downstream of *sinI*, *sinR* is transcribed constitutively and independently (20). The activity of SinR, a master regulator of biofilm matrix production, is regulated by two antagonists, namely, SinI and SlrR, that can form alternative complexes with SinR, preventing the formation of SinR_4_ (the active tetramer form of SinR). On the one hand, the SinI dimer (SinI_2_) interacts with the SinR dimer (SinR_2_) and forms an SinI-SinR heterodimer (SinI·SinR) (21). On the other hand, SlrR dimer (SlrR_2_) associates with SinR_2_ and forms an SlrR_2_·SinR_2_ heterotetramer (21–23). The expression of *slrR* is also repressed by SinR_4_, thereby resulting in a double-negative feedback loop between SinR and SlrR. This double-negative feedback loop between SinR and SlrR forms a bistable switch and controls matrix production and cell chaining (24, 25). In this SinI-SinR-SlrR network, Spo0A~P concentration serves as the input by directly controlling SinI expression. Therefore, the Spo0A and SinI-SinR-SlrR network systems precisely control the level of SinR during growth and starvation conditions. During growth under nutrient-rich conditions, little if any Spo0A~P is present, and thus, the Spo0A-controlled SinI, an antagonist of the SinR transcription factor, is not expressed highly. As a result, the constitutively expressed SinR represses a set of genes and operons involved in biofilm matrix production, including the *tapA* operon, allowing cells to grow (26). Upon starvation, relatively low concentrations of Spo0A~P generated via the phosphorelay activate the expression of SinI (27), which in turn sequesters and thereby antagonizes SinR (17, 18). SinR is also sequestered by forming a SlrR_2_·SinR_2_ heterotetramer during this period (21). When starvation persists, cellular concentrations of Spo0A~P further increase with the decrease in cell growth rate and directly stimulate the expression of genes involved in sporulation (13, 16, 28).

Intriguingly, despite being both activated by Spo0A~P, sporulation and matrix production appear mutually exclusive, as the matrix production drops significantly in cells initiating sporulation (24, 29). As a result, the population-average biofilm matrix production level decreases at the late stages of starvation (24). These observations are attributed to the repression of SinI expression by high Spo0A~P levels and to the effect of sporulation initiation on the gene dosage of *sinR* and *slrR* (24). However, our recent results showed that artificially induced high Spo0A~P levels cannot repress the expression of matrix production genes, which questions the explanation that high Spo0A~P levels and sporulation repress matrix production (11). Therefore, the mechanisms of cell fate determination at late stages of starvation are still not fully understood.

Understanding cell fate decision in the biofilm matrix production is possible only on the level of individual B. subtilis cells since the expression of biofilm matrix genes is highly heterogeneous (30, 31). This heterogeneity can result from variability in gene expression due to changes in global physiological parameters (extrinsic noise) and fluctuations at the level of individual genes amplified by local gene regulatory mechanisms (intrinsic noise). Our recent work showed that heterogeneity in matrix production is regulated through the effects of two different kinases (11). In particular, we showed that KinC reduces single-cell heterogeneity of Spo0A~P resultant from the extrinsic noise in cellular growth rate and thereby increases the fraction of cells that activate matrix production (11). However, the results also suggest that noise in growth rate is not sufficient to fully explain the single-cell distribution of matrix-production gene expression (11).

In this work, using stochastic modeling, we investigate how the extrinsic noise in growth rate and intrinsic noise in the SinI-SinR-SlrR network affect the distribution of matrix-producing cells at different times poststarvation. Furthermore, we use our models to uncover the competing effects of the slowdown of growth rate on the SinI-SinR-SlrR network. This model is used to explain the dynamics of biofilm matrix production under different genetic perturbations and to investigate why biofilm matrix production and sporulation appear mutually exclusive on a single-cell level. Experimental tests of the model predictions confirm the proposed mechanisms of cell-fate control.

## RESULTS

### An increase in Spo0A~P is necessary but not sufficient for matrix production.

Previously, assuming a deterministic relationship between Spo0A~P levels and *tapA* expression, we failed to quantitatively match the single-cell distribution of matrix production (11). In particular, the model predicted that the fraction of matrix-producing cells is higher than the experimentally observed fraction (11). Since the fluctuations in the SinI-SinR-SlrR network are known to affect matrix production (25, 30), we hypothesized that stochastic properties of the SinI-SinR-SlrR network that controls the relationship between Spo0A~P levels and *tapA* expression could reduce the fraction of matrix-producing cells. To test this hypothesis, we constructed a detailed mathematical model of this network and used it to examine the relationship between Spo0A~P levels and TapA reporter concentration in deterministic and stochastic simulations.

The schematics of the modeled network shown in Fig. 1A include transcriptional and posttranslational interactions between SinI, SinR, and SlrR (see Materials and Methods for details). However, the significance of the double-negative feedback loop between SinR and SlrR on matrix production is unclear. To answer this question, we first investigated how the steady-state concentration of TapA is affected by the Spo0A~P level via the SinI-SinR-SlrR network. As Fig. 1B shows, our model with the deterministic simulations demonstrated that, when Spo0A~P level is low, the system has only one stable steady state (Fig. 1B, blue solid line) in which *tapA* expression is not activated and TapA concentration is low. When the Spo0A~P level is higher than a threshold, a matrix-production-on steady state appears (Fig. 1B, red solid line) and the system shows bistability. If we further increase Spo0A~P concentration (>0.4 μM), the steady-state value of TapA would not change much (Fig. 1B, red solid line). This result can be explained in that the expression of SinI requires only a low threshold of Spo0A~P (10); when Spo0A~P goes higher, the expression of SinI would be saturated and cannot be further increased. Thus, the activation of Spo0A~P is necessary but not sufficient for matrix production; when the Spo0A~P level is lower than the threshold, the *tapA* cannot be activated, and when the Spo0A~P level is higher than the threshold, both the high and the low *tapA* expression states are present. In the latter regime, the expression is hysteretic in our deterministic simulations, i.e., cells starting with a high level of *tapA* expression remain high, whereas cells starting with a low level will remain low.

To test the possibility of switching between steady states, we constructed a stochastic version of the network (see Materials and Methods for details) and conducted a stochastic simulation of the model for fixed low (0.05 μM) and high (1 μM) Spo0A~P levels, i.e., in the monostable and bistable conditions for the deterministic model. As Fig. 1C shows, for a high Spo0A~P concentration, the *tapA* expression could be activated in a stochastic manner (red line); whereas, for low a Spo0A~P concentration, the *tapA* expression remains around the low level (blue line). The results demonstrated that fluctuations of the SinI-SinR-SlrR network can lead to stochastic activation of *tapA* expression but only if Spo0A~P is sufficiently high. In other words, high Spo0A~P is necessary but not sufficient for *tapA* expression.

### Extrinsic noise in growth rate and intrinsic noise in the SinI-SinR-SlrR network can explain the individual-cell distribution of matrix gene expression.

**FIG 2:**
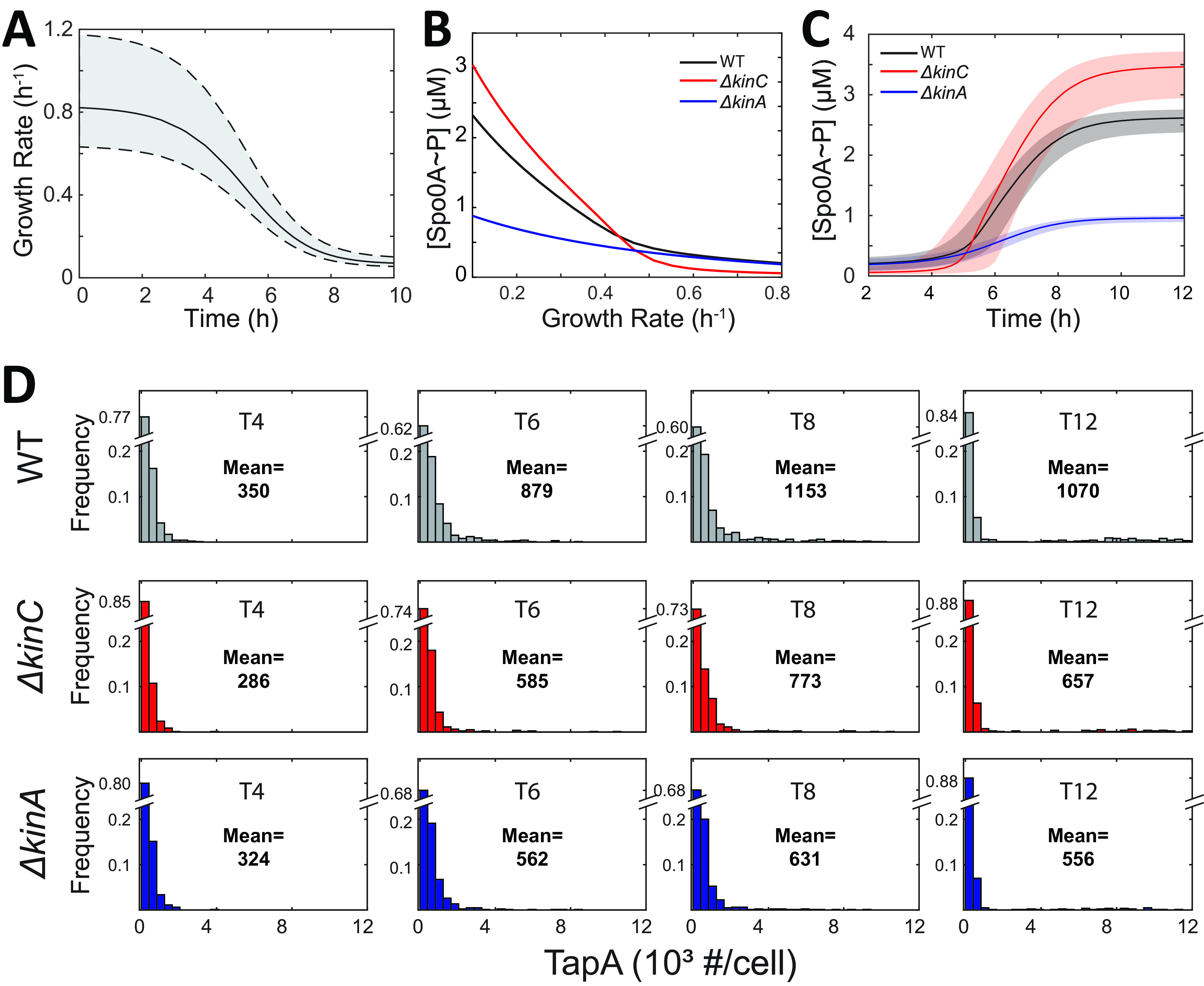
Heterogeneity of *tapA* expression is controlled by growth rate and the SinI-SinR-SlrR network. (A) The dynamics and fluctuation (shaded area shows ± *σ* region) of growth rate (*y* axis) over time (*x* axis) were predicted by a Moser-type model (34). (B) Model-predicted Spo0A~P concertation (*y* axis) as a function of the growth rate (*x* axis) in the WT, Δ*kinC*, and Δ*kinA* strains. (C) Predicted Spo0A~P dynamics and fluctuations as a function of time (shaded area shows ± *σ* region) in the WT, Δ*kinC*, and Δ*kinA* strains. (D) Prediction of single-cell distribution of *tapA* expression at T4, T6, T8, and T12 (i.e., 4 h, 6 h, 8 h, and 12 h, respectively) in the WT, Δ*kinC*, and Δ*kinA* strains using the results of C as an input to the stochastic model of the SinI-SinR-SlrR network. Population mean levels of *tapA* expression (number of TapA molecules/cell) are indicated in each panel. Note that the maximum value for the first bin is indicated in the broken *y* axis with the same scale for the remaining bins used in each panel.

Spo0A~P levels increase with the slowdown of cell growth rate during nutrient starvation (15, 28, 32, 33). Thus, next, we investigated whether the combination of the intrinsic noise in the SinI-SinR-SlrR network and the fluctuations of growth rate can explain the single-cell-level heterogeneity of *tapA* expression. To reproduce the single-cell-level distribution of *tapA* expression in a starving community, following our previous work (11), we used a Moser-type model (34) to describe the dynamics of the population-average growth rate (Fig. 2A, solid line). We also assumed that the cell generation time follows a normal distribution with a coefficient of variation (CV) of 0.25. The shaded area in Fig. 2A shows the range of the growth rate in ~70% of cells (±*σ*). It was shown that the distribution of single-cell Spo0A~P concentrations could be sufficiently explained by the fluctuations in the growth rate (15). Thus, we assumed that the Spo0A~P level is determined by the growth rate, so the noise in the Spo0A~P level originates fully from the growth rate fluctuations. Using the same model in our previous work (11), we predicted how the Spo0A~P levels change with the growth rate in the wild-type (WT) strain and the strain harboring the deletion of *kinA* (Δ*kinA*) and *kinC* (Δ*kinC*) that were shown to affect *tapA* expression dynamics (Fig. 2B). Based on the dynamics and fluctuation of the growth rate, we modeled the dynamics and distribution of Spo0A~P levels in different strains (Fig. 2C).

Furthermore, to investigate the single-cell-level heterogeneity of *tapA* expression, we performed stochastic simulations of the SinI-SinR-SlrR network for different cell lineages in parallel (see Materials and Methods for details). To this end, Spo0A~P levels were used as the input and were sampled from the predicted distribution. Then, we calculated the distribution of *tapA* expression levels at different times (after 4, 6, 8, and 12 h of culture, denoted as T4, T6, T8, and T12, respectively) in each of the three strains (WT, Δ*kinA*, and Δ*kinC*). By comparing the results of the model (Fig. 2D) with the experimental data (see Fig. S1 in the supplemental material) from our previous work (24), we can conclude that the shape of predicted distributions qualitatively matches those experimentally measured. Importantly, unlike the model that did not explicitly account for bistability and intrinsic noise in the SinI-SinR-SlrR network (11), our new model correctly predicts that the majority of cells have very low *tapA* expression (the first bin of the histogram in two-dimensions [2D]). The fraction of cells with very low *tapA* expression (the first bin) is correctly predicted to decrease with time and to be larger in the Δ*kinC* strain than in the WT strain. Furthermore, the model also predicts the fraction of cells that do not activate matrix production to be smaller in the Δ*kinA* strain than in the WT. Therefore, the results shown in Fig. 2 suggest that changes in the *tapA* expression distributions in single cells are changed with time and genetic perturbations can be explained through the effects of noise in growth rate and in the gene expression of SinI-SinR-SlrR network. Thus, the model suggests the mechanisms behind the observed dynamics.

10.1128/msystems.00622-22.1 FIG S1 The experimentally observed distribution of *tapA* expression. The distribution of mean fluorescence intensity of *PtapA-GFP* in WT and Δ*kinC* cells at T4, T6, and T8. The value of the first bin was labeled on each histogram. Reproduced using data from reference 11. Download FIG S1, PDF file, 0.6 MB. Copyright © 2023 Chen et al. 2023 Chen et al. https://creativecommons.org/licenses/by/4.0/ This content is distributed under the terms of the Creative Commons Attribution 4.0 International license.

To understand how the fraction of matrix-producers changes with time, we note that under starvation conditions, the average cellular growth rate decreases with time (Fig. 2A) and, as a result, based on the previously developed models of phosphorelay (15), cellular Spo0A~P levels increase with time for all the strains (Fig. 2C). However, as predicted by our model (Fig. 1), the activation of Spo0A is a permissive signal but not an instructive one for matrix production. Thus it is the intrinsic noise of the SinI-SinR-SlrR network ensures that cells transition to the matrix-expressing state. A decrease in the fraction of cells not expressing *tapA* with time (Fig. 2D, WT [T4 to T8], and note the change in the *y* axis scale in each panel) can be explained by (i) the increase in the number of cells with sufficient Spo0A~P and (ii) the increased chance that gene expression fluctuation of sufficient magnitude to activate the SinI-SinR-SlrR switch will occur. In support of the latter mechanism, our simulation showed that the single-cell distribution of *tapA* expression is not in a steady state; when we fixed the growth condition at T6 and ran the simulation for a longer time, the fraction of *tapA*-expressing cells would increase further (see Fig. S5 in the supplemental material). Notably, the mean level of *tapA* expression and the fraction of cells that activated *tapA* in our model are predicted to be lower at T12 than those at T8 for all the strains. This effect is observed even though we do not introduce the repression of *sinI* by high levels of Spo0A~P in our model. The mechanism of this decrease is investigated in the next section.

10.1128/msystems.00622-22.5 FIG S5 *tapA* expression is not in a steady state during simulation. The red and black line shows the dynamics of mean TapA levels of WT and Δ*kinC* strains, which are the same as those in Fig. 4B. The green line shows the mean TapA levels of Δ*kinC* strain for simulations assuming the growth rate stops decreasing at T6 and is fixed thereafter. Download FIG S5, PDF file, 0.6 MB. Copyright © 2023 Chen et al. 2023 Chen et al. https://creativecommons.org/licenses/by/4.0/ This content is distributed under the terms of the Creative Commons Attribution 4.0 International license.

To understand the effects of KinA and KinC on matrix production, we note that in the Δ*kinA* strain, Spo0A~P level is lower than in the WT strain (Fig. 2C, after T5), so the probability of expressing *tapA* is also lower in the *ΔkinA* strain than that in the WT strain (Fig. 2D, WT versus Δ*kinA*, note the change in *y* axis scale in each panel). In the Δ*kinC* strain, the Spo0A~P level was lower than in the WT strain at early times, such as T4 (Fig. 2C), so the probability of expressing *tapA* was also lower in the Δ*kinC* strain than in the WT strain (Fig. 2D, note the change in *y* axis scale in each panel). These effects can all be explained by a smaller fraction of cells with a Spo0A~P level above the permissive threshold. However, our previous work (11) demonstrated that at later times KinC acts as a phosphate-group sink and its deletion would increase Spo0A~P at a later time (Fig. 2C). Nevertheless, the fraction of cells not expressing *tapA* remains lower in the Δ*kinC* strain at T6 to T12. This effect is related to the ability of KinC to reduce single-cell heterogeneity of Spo0A~P due to fluctuations of cell growth (extrinsic noise) investigated in our previous publication (11).

### Slowdown of cellular growth has two opposing effects on the SinI-SinR-SlrR network.

Our model shows the decrease of *tapA* expression at T12 for all the strains considered (Fig. 2D, T12). For wild-type cells, this result is consistent with previous experimental observations (24). We, therefore, set to understand the mechanisms of the decrease in *tapA* expression at late times in our model. A previous study suggested that the observed decrease of *tapA* expression under prolonged starvation is associated with (i) the decreased levels of SinI due to the direct repression of *sinI* by high levels of Spo0A~P and (ii) increased levels of SinR over SlrR due to changes in gene dosage during sporulation (24). Both mechanisms result in the increased active SinR (SinR_4_ tetramer), leading to the repression of *tapA* expression. However, in our model, neither the repression of SinI expression by high Spo0A~P levels nor the sporulation process was explicitly included. Instead, gene dosages and protein concentrations change as a function of cellular growth rate. This finding indicates the slowdown of the growth rate can somehow negatively regulate *tapA* expression via the SinI-SinR-SlrR network.

**FIG 3:**
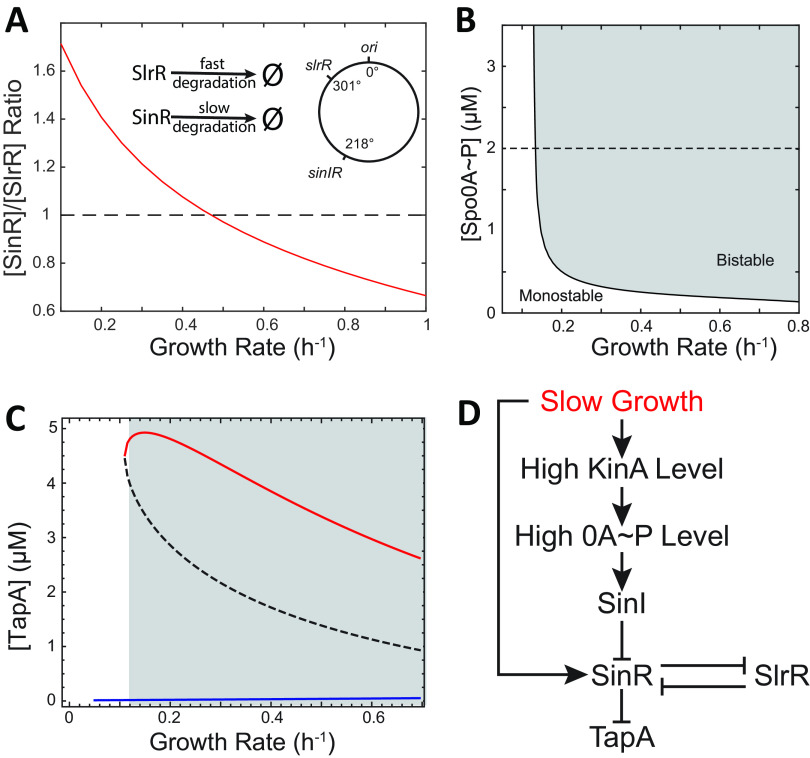
The behavior of the SinI-SinR-SlrR network is determined by both the Spo0A~P level and the growth rate. (A) Changes in the predicted ratio of the total concentrations of SinR to SlrR as a function of growth rate in the absence of transcriptional regulation. The inset illustrates degradation rates of SlrR and SinR and their gene positions on the chromosome. (B) Bifurcation between mono- (clear) and bistability (shaded) of *tapA* expression state controlled by Spo0A~P level (*y* axis) and growth rate (*x* axis) via the SinI-SinR-SlrR network. (C) High (red) and low (blue) steady states of TapA concentration as a function of growth rate at the fixed 2 μM Spo0A~P condition as indicated with the dashed line in B. The shaded region shows a bistable region in which a high TapA-expressing state is possible (red). Decrease of the growth rate outside the bistable region lead to switch into deactivated *tapA* expression state (blue line). (D) The proposed feed-forward network showing how growth rate regulates biofilm matrix production via the SinI-SinR-SlrR network.

In our model, growth rate affected cellular protein concentrations mainly by affecting their dilution rate. For stable proteins like SinR, the effective degradation rate (the sum of the degradation rate and dilution rate caused by growth) is dominated by the dilution rate, and the slowdown of growth will lead to increases in their concentrations. In contrast, SlrR is known to be quickly degraded *in vivo* (35), so the change in growth rate has a relatively small effect on the effective degradation rate (Fig. 3A). In addition, our model suggested that the SinR/SlrR ratio is controlled by the gene dosage effect related to the position of the genes on chromosomal DNA (24), as was the case for KinA and Spo0F (14, 15, 32). In general, gene distance from the origin of chromosome replication (*oriC*) influences gene copy number in a periodical manner during the growth cycle of a bacterial cell (36). During DNA replication (C-period) (see Fig. S2A in the supplemental material), genes proximal to *oriC* (ori) are replicated first and will have a higher gene dosage relative to the genes proximal to the replication terminus (*ter*) (Fig. S2B and C). After replication is complete, the gene dosage returns to a 1:1 ratio. A slowdown of growth has a greater effect on the cell cycle period than on the C-period and thereby increases the duration of the period during which there is no active DNA replication, resulting in a 1:1 gene dosage (Fig. S2). Therefore, cells growing slower are expected on average to have less excess in gene dosage for ori-proximal genes. In the case of the SinI-SinR-SlrR network, *slrR* is located at the origin proximal region, while *sinI* and *sinR* genes are at the origin distal region. Thus, when cells grow rapidly, the dosage of *slrR* exceeds those of *sinI* and *sinR* for a longer part of a cell cycle leading to a higher *slrR* production rate. However, when the growth rate slows down, cell cycle-averaged excess gene dosage for *slrR* is smaller than that when the growth rate is fast; the relative production rate of *sinR* to *slrR* increases with growth slowdown. In summary, different protein degradation rates and different gene positions cause the ratio of SinR and SlrR to increase with a decreasing growth rate (Fig. 3A).

10.1128/msystems.00622-22.2 FIG S2 Growth rate affects gene dosage for the replication-terminus proximal genes. (A) The duration of the C period (replication) and the whole-cell cycle as functions of growth rate. (B and C) The change of the copy numbers of a replication-terminus proximal gene (*P = *1) and a replication-origin proximal gene (*P = *0) within a cell cycle. The copy numbers were plotted for growth rates equal to 0.6 h^−^^1^ (B) and 0.15 h^−^^1^ (C). For a gene proximal to the replication origin, the gene dosage is 2 during the whole-cell cycle regardless of the growth rate. For a gene proximal to the replication terminus, the fraction of time that the gene dosage is equal to 2 is larger when the growth rate is lower. Download FIG S2, PDF file, 0.7 MB. Copyright © 2023 Chen et al. 2023 Chen et al. https://creativecommons.org/licenses/by/4.0/ This content is distributed under the terms of the Creative Commons Attribution 4.0 International license.

To understand this effect in our model, we first used a deterministic model of the SinI-SinR-SlrR network and computed a bifurcation diagram, i.e., the plot that illustrates how the region of bistability changes in response to independently changing Spo0A~P concentration and the growth rate (Fig. 3B). The result indicated that bistability (and existence *tapA* expression state) requires the Spo0A~P level to be higher than a threshold (as we saw in Fig. 1B) and the growth rate to be not too slow (shaded region in Fig. 3B). If we fixed Spo0A~P at a relatively high level (Fig. 3B, dashed line), the system is bistable but only at high growth rates (Fig. 3C). As growth slows down, there will be an insufficient amount of SinI and SlrR to fully inhibit the activity of SinR (Fig. 3A). As a result, the system would enter the monostable region and the matrix-production-on state (Fig. 3C, red solid line) would disappear, i.e., *tapA* will be repressed. Stochastic simulation under fixed growth rates and Spo0A~P levels confirmed that bistability only exists when both the Spo0A~P level and the growth rate are high enough (see Fig. S3 in the supplemental material).

10.1128/msystems.00622-22.3 FIG S3 The distribution of SlrR and SinR levels at different Spo0A~P levels and growth rates from stochastic simulations. Download FIG S3, PDF file, 0.7 MB. Copyright © 2023 Chen et al. 2023 Chen et al. https://creativecommons.org/licenses/by/4.0/ This content is distributed under the terms of the Creative Commons Attribution 4.0 International license.

In light of these results, we propose that the growth rate affects *tapA* via two opposing mechanisms acting on the SinI-SinR-SlrR network (Fig. 3D). On the one hand, by regulating the upstream phosphorelay network, the slowdown of growth rate raises the Spo0A~P level via increasing the KinA level (15) and thereby activates SinI expression (27). This process would lead to sequestration of SinR into the SinI·SinR complex and lead to derepression of *tapA*. On the other hand, the slowdown of growth directly increases the relative concentration of SinR to SlrR, decreasing the amount of SinR sequestered in the SlrR_2_·SinR_2_ complex. This effect leads to an increase in the free and active SinR_4_ form, resulting in the repression of *tapA*. In other words, there is an incoherent feed-forward loop between the growth slowdown and *tapA* expression, and this motif is known to produce nonmonotonic dynamics of gene expression (37, 38).

Note that, experimentally, Spo0A~P concentration and the growth rate are not independent and a slowdown of growth results in the increase of Spo0A~P (15). Explicitly testing the above incoherent feed-forward hypothesis, therefore, requires data on genetic perturbations that affect the Spo0A~P concentration, e.g., those affecting phosphorelay and perturbations affecting growth dynamics (i.e., how cell growth rate changes with time).

### Slowdown of growth rate directs the decrease of matrix gene expression at late stages of growth.

To validate the above-proposed model that the slowdown of growth rate is the main reason for the decrease of *tapA* expression at late times, we predicted the dynamics of *tapA* expression levels under different genetic perturbations that change the dynamics of Spo0A~P levels. To this end, in addition to the WT and the strains harboring the deletion of two phosphorelay kinases (*ΔkinA* and *ΔkinC*) considered in Fig. 2, we also investigated the effects of Sda, an inhibitor of KinA by forming an inactive complex Sda-KinA (39). Deletion of *sda* (Δ*sda*) is expected to raise Spo0A~P levels as a result of increased KinA activity, leading to promoted sporulation (39, 40), whereas deletion of *kinA* (*ΔkinA*) will do the opposite since it is the major sporulation kinase (9, 12). Given the ability of KinC to serve as a phosphate sink, i.e., by removing a phosphoryl group from Spo0A~P, we expect that the Δ*kinC* strain would display higher Spo0A~P levels (Fig. 2C) and sporulation frequencies (11). Therefore, investigating these strains may allow us to separate the effects of Spo0A~P concentration and the growth rate on matrix production.

**FIG 4:**
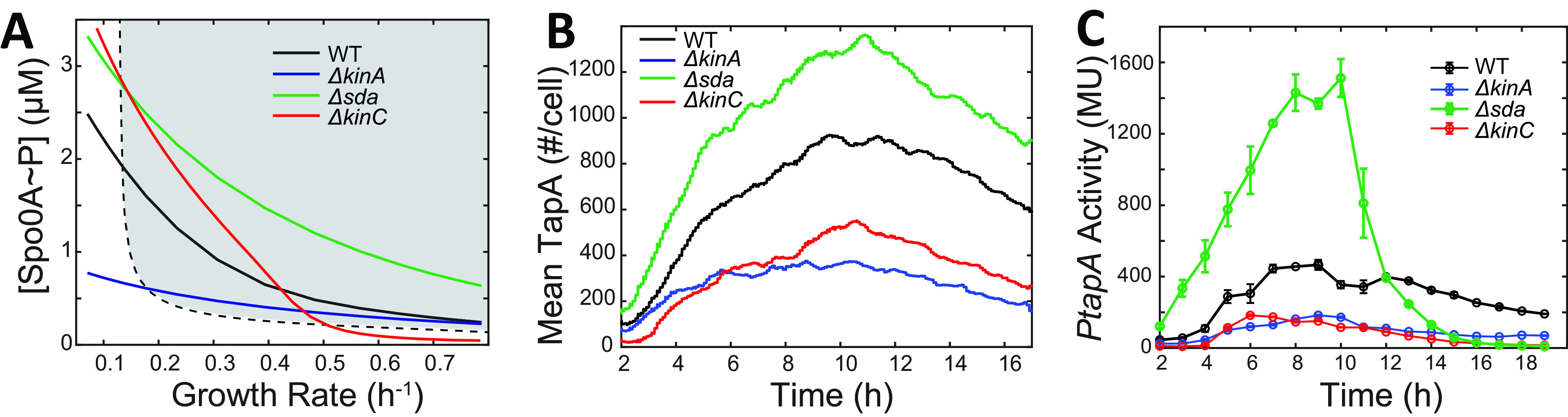
Repression of *tapA* expression caused by a slowdown of growth rate. (A) Predicted changes in Spo0A~P concentration as a function of growth rate in the WT, Δ*kinA*, Δ*kinC*, and Δ*sda* strains superimposed on Fig. 3B displaying bifurcation (dashed line) between mono- (clear) and bistability (shaded) of *tapA* expression state controlled via the SinI-SinR-SlrR network. (B) Stochastic simulation of *tapA* expression dynamics in the WT, Δ*kinA*, Δ*kinC*, and Δ*sda* strains. The *x* and *y* axis indicate time (h) and population-averaged (mean of *n* = 1,000 simulations) *tapA* expression levels (number of molecules/cell), respectively. (C) Experimentally measured *tapA* expression at a population level in the WT, Δ*kinA*, Δ*kinC*, and Δ*sda* strains. Culture samples were collected at the indicated times (*x* axis) after the start of incubation and assayed for β-galactosidase activity from *PtapA-lacZ* (Miller units [MU], *y* axis). The mean activities of three independent experiments are shown with standard deviations as error bars.

Using a deterministic model of phosphorelay from our prior work (11, 14, 32), we determined Spo0A~P concentration as a function of the growth rate in each of the above strains. As Fig. 4A shows, for all of the strains, Spo0A~P increased with a decrease in growth rate. As expected, Spo0A~P levels in the WT strain were higher than in those in the Δ*kinA* strain but lower than those in the Δ*sda* strain. In the Δ*kinC* strain, Spo0A~P levels were lower than in the WT strain under high growth rates at early times of culture. However, Spo0A~P levels became higher in the Δ*kinC* strain than in the WT strain under low growth rates at later times of culture. Superimposing these trajectories on the bifurcation diagram, one can note that all of these values left the bistable region at around the same value of growth rate, due to the bistability region boundary being nearly vertical (Fig. 4A, dashed line). Therefore, we expect that *tapA* expression would decline at around the same growth rate in all three strains despite distinct that Spo0A~P dynamics can be seen in each of them. Assuming that growth-rate dynamics are the same for all strains (11), we can use the stochastic simulation of the model to predict *tapA* expression. The results predicted that in all of the strains, *tapA* expression decreased at about the same time (~10 h) (Fig. 4B). Alternatively, if the hypothesis that the high Spo0A~P levels leading to sporulation drive the decrease of *tapA* expression is correct (24), our simulations would expect very different times of the peaks of *tapA* expression in those strains (see Fig. S4 in the supplemental material). Critically, in the Δ*kinA* strain, since the Spo0A~P level would not go beyond the sporulation threshold and thus the sporulation efficiency is very low (12), we predicted the decrease of *tapA* expression to happen later (Fig. S4).

10.1128/msystems.00622-22.4 FIG S4 The traditional explanation of why *tapA* expression decreases at late times. (A) The traditional model of how high Spo0A P levels repress *tapA* expression. On the one hand, high Spo0A~P levels repress the expression of SinI. On the other hand, high Spo0A~P levels trigger sporulation, which changes the dosage between SinR and SlrR and eventually represses the expression of *tapA*. (B) The Spo0A~*P* values as functions of growth rate in WT, Δ*kinA*, and Δ*sda* strains. The assumed threshold of Spo0A~P level that is sufficient to repress *tapA* expression is shown as the dashed line. (C) The predicted dynamics of *tapA* expression with the traditional model in WT, Δ*kinA*, and Δ*sda* strains. Download FIG S4, PDF file, 0.7 MB. Copyright © 2023 Chen et al. 2023 Chen et al. https://creativecommons.org/licenses/by/4.0/ This content is distributed under the terms of the Creative Commons Attribution 4.0 International license.

To test the prediction that the decrease of *tapA* expression occurs simultaneously in all strains (WT and deletion mutants), we performed experimental measurements of *tapA* expression dynamics in those strains with a β-galactosidase (β-gal) reporter. In the results shown in Fig. 4C, the peak of *tapA* expression occurred at 8 to 10 h in the tested strains except for the Δ*kinC* strain where the peak is slightly earlier (6 to 7 h). Notably and similarly to the other strains tested, the decrease in *tapA* expression after 9 to ~10 h was observed in the Δ*kinA* strain where little sporulation is triggered (Fig. 4C). These results support our hypothesis that the decrease of *tapA* expression is due to the slowdown of growth rate, rather than the high Spo0A~P levels or the sporulation. Thus, these data are qualitatively consistent with the idea that the incoherent feed-forward-loop mechanism through the SinI-SinR-SlrR network is important to control *tapA* expression.

Even though our model supports general trends in the observed *tapA* dynamics, the experimental results in the Δ*kinC* strain showed that the *tapA* expression peaked at around 6 to 7 h and then decreased earlier than in the other strains (Fig. 4C). These experimental data were slightly different from the modeling data (Fig. 4B). Moreover, the experimental data showed that, in the Δ*sda* strain, the *tapA* expression decreases much more rapidly (Fig. 4C) than predicted (Fig. 4B). These inconsistencies may be due to the early onset of sporulation in the Δ*kinC* and Δ*sda* strains (11). Spore formation may repress the σ^A^-dependent *tapA* expression and/or interfere with β-galactosidase activities due to the reduced σ^A^ activity during sporulation (41). These effects were not considered in the model.

To further understand the factors contributing to the decrease of *tapA* expression in population-average assays, we used our model to calculate the fraction of *tapA*-expressing cells and the mean TapA level of the expressing cells (see Fig. S7 in the supplemental material). Due to the global effects of a growth slowdown, the mean TapA level of *tapA*-expressing cells increases with time in all the strains (Fig. S7C). The fraction of *tapA*-expressing cells (Fig. S7B) shows a similar trend as the overall *tapA* expression level (Fig. S7A), except that the peak of the *tapA*-expressing cell fraction comes earlier than the overall *tapA* expression. These results show that the decrease of *tapA* expression at late times and the differences in TapA dynamics in different strains are affected mainly by the change of the *tapA*-expressing cell fraction; meanwhile, the increase of TapA level in *tapA*-expressing cells compensates for the decrease of the *tapA*-expressing cell fraction and postpone the decrease of the overall *tapA* expression level.

10.1128/msystems.00622-22.7 FIG S7 Effects of *tapA*-expressing cell fraction and *tapA*-expressing levels. (A) Simulated *tapA* expression dynamics in different strains (copied from Fig. 4B). (B) Simulated dynamics of the fraction of *tapA*-expressing cells. (C) Simulated dynamics of mean TapA level of all *tapA*-expressing cells. Download FIG S7, PDF file, 0.1 MB. Copyright © 2023 Chen et al. 2023 Chen et al. https://creativecommons.org/licenses/by/4.0/ This content is distributed under the terms of the Creative Commons Attribution 4.0 International license.

### Perturbation to cell growth rate alters matrix gene expression dynamics.

**FIG 5:**
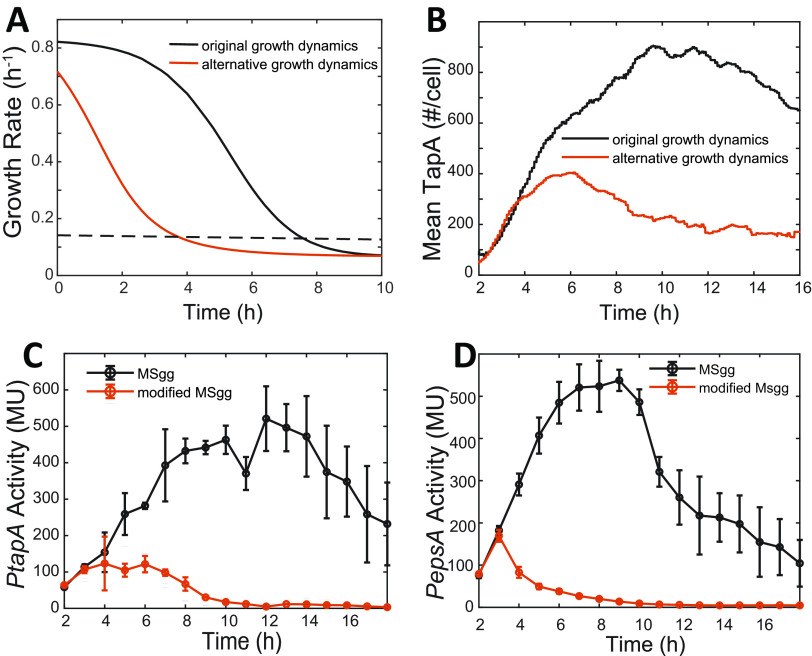
The dynamics of *matrix* gene expression under different growth dynamics. (A) Stochastic simulation of growth rate (*y* axis) as a function of time (*x* axis) under normal (black) and slow (red) growth conditions. When different growth dynamics depicted in A were used as an input in our stochastic simulations, the model predicted stochastic simulation of *tapA* expression (mean number of TapA molecule per cell, *y* axis) as a function of time (*x* axis) under normal (black) and slow (red) growth conditions (B). (C, D) Experimentally measured expression of *tapA* (C) and *epsA* (D) at a population level in the WT cells grown in normal (black) and nitrogen-reduced (reduced to 1/10 of the normal level, red) MSgg media. Culture samples were collected at the indicated times (*x* axis) after the start of incubation and assayed for β-galactosidase activity from *PtapA-lacZ* (Miller units [MU], *y* axis). The mean activities of three independent experiments are shown with standard deviations.

The results thus far indicated that perturbing the Spo0A~P dynamics does not significantly affect the time when *tapA* expression peaks. However, if our hypothesis is correct that the slowdown of growth rate is the main reason for the decrease of *tapA* expression, we expect that a change in growth dynamics would shift the time of *tapA* expression peak. To demonstrate this shift with our model, we used alternative growth rate dynamics as inputs to our model (Fig. 5A). Stochastic simulation of the model predicted that if the growth rate slows down more rapidly (Fig. 5A, red curve), the *tapA* expression would start to decrease earlier and the resulting maximum *tapA* expression level would also be significantly lower than the unperturbed cell growth rate dynamics (Fig. 5B).

To test this prediction experimentally, we artificially changed cell growth rate by reducing the nitrogen source (diluted glutamate by 10 times) in minimal salts glycerol glutamate (MSgg) medium (modified MSgg medium, see Materials and Methods). Under culture conditions in the modified MSgg medium, the nitrogen source would be depleted faster and, therefore, cell growth would slow down earlier than in the original MSgg medium. As Fig. 5C shows, in the cells grown in the modified MSgg medium, the decrease of *tapA* expression happens much earlier and the peak value becomes much lower than in the cells grown in the original MSgg, which is consistent with the model prediction (Fig. 5B). To further verify that the slowdown of growth rate is the main reason for the decrease of matrix gene expression, we examined another matrix gene, *epsA*, which is also under the control of the SinI-SinR-SlrR network. As shown in Fig. 5D, the trends of *epsA* expression under two different conditions were qualitatively similar to those of the *tapA* expression. These results further support our hypothesis that the slowdown of growth is the primary reason for the decrease of matrix gene expression at the late stages of starvation.

### Slowdown of cell growth leads to mutually exclusive cell fates.

**FIG 6:**
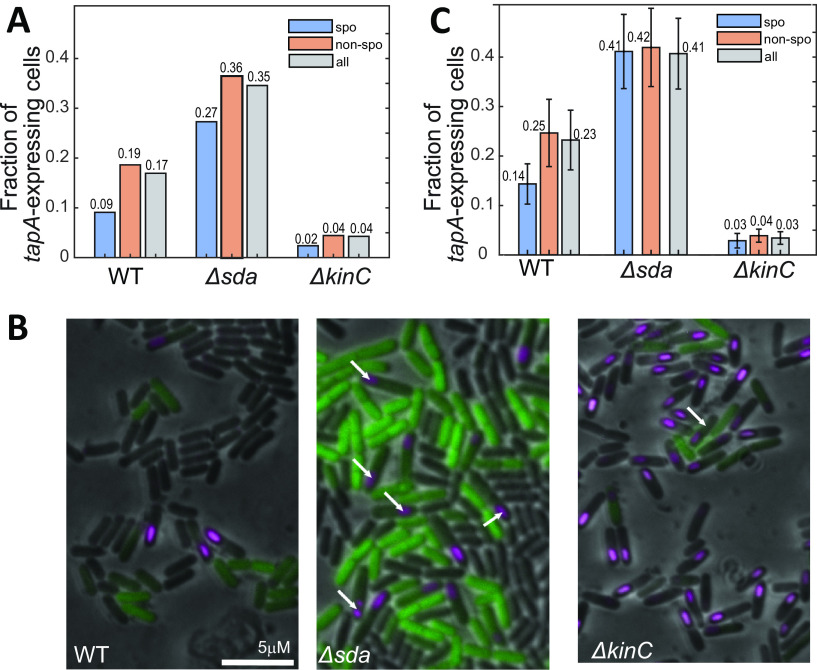
Acceleration of Spo0A~P dynamics decrease mutual exclusiveness of sporulation and matrix production. (A) Stochastic simulation of *tapA* expression in sporulating cells (spo; slow growth); nonsporulating cells (non-spo; fast growth); and all cells in the WT, Δ*sda*, and Δ*kinC* strains. (B) Fluorescent images of the WT, Δ*sda*, and Δ*kinC* cells harboring both the GFP-LCN (unstable GFP) reporter under the control of *tapA* promoter (*PtapA-gfp-lcn*) and the mCherry reporter under the control of the *spoIIQ* promoter (*PspoIIQ-mCherry*). Cells were cultured in MSgg medium and processed for imaging at 8 h after the start of culture. Cells displaying both GFP (*tapA* expression) and mCherry (*spoIIQ* expression for sporulation) are indicated with arrows. Scale bar: 5 μm. (C) The experimentally measured fraction of cells expressing *tapA* in sporulating (spo) cells; nonsporulating (non-spo) cells; and all cells in WT, Δ*sda*, and Δ*kinC* strains. Error bars indicate standard deviation for *n* = 9 images taken from 3 independent cultures for each strain.

Since our model explains the population-level decrease of *tapA* expression at late times, we hypothesized that it can also explain why sporulation and biofilm matrix production appear mutually exclusive on a single-cell level. To test this hypothesis, we predicted the *tapA* expression and sporulation levels in single cells of the WT, Δ*sda*, and Δ*kinC* strains, using stochastically various but on average slowing cell growth dynamics (Fig. 2A) as an input. Following our previous study (16), cells displaying high-threshold Spo0A~P levels were considered sporulating. The fraction of *tapA*-expressing cells between sporulating (spo) and nonsporulating (non-spo) cells at T8 was then calculated by simulation (Fig. 6A). These results showed that, in the WT strain, about 19% (0.19) of nonsporulating (non-spo) cells activate *tapA* expression, but this fraction is only 9% (0.09) for sporulating cells (spo) (Fig. 6A). These results were qualitatively consistent with the published experimental results of reference 24, which indicates that our model can also explain single-cell distributions of matrix production and sporulation. Thus, our model predicts that mutual exclusiveness can be explained by the repression of matrix production at the low growth rate at which WT cells initiate sporulation.

Furthermore, we can use our model to predict how genetic perturbation in phosphorelay affects mutual exclusiveness. Notably, our results indicated that the fraction of *tapA*-expressing cells increased more in the Δ*sda* strain than that in the WT strain (0.35 versus 0.17) (Fig. 6A). However, when the increase in *tapA*-expressing cells in the Δ*sda* versus WT strains is broken down by sporulation status, we note that this increase was larger in the sporulating cells (~3-fold, from 0.09 to 0.27) than in nonsporulating cells (less than 2-fold, from 0.19 to 0.36) (Fig. 6A). On the other hand, in the Δ*kinC* strain, the fraction of *tapA*-expressing cells was much lower than in the WT strain (0.17 versus 0.04) (Fig. 6A). This decrease in the fraction of *tapA*-expressing cells is about the same fold in the sporulating cells (~4-fold) and in nonsporulating cells (~4.3-fold) (Fig. 6A). These simulation results predict that the deletion of *sda* (Δ*sda*) not only increases the overall fraction of *tapA*-expressing cells but also increases the fraction of the cells that both activate *tapA* expression and sporulation. On the other hand, the deletion of *kinC* (Δ*kinC*) decreases the fraction of *tapA*-expressing cells among sporulating cells, as well as all the cells. In other words, the deletion of *sda* (Δ*sda*) would “weaken” the apparent mutual exclusiveness of matrix production and sporulation, while the deletion of *kinC* (Δ*kinC*) would affect it only slightly.

To verify these predictions, we experimentally measured *tapA* expression (with *PtapA*-GFP) and sporulation (with forespore-specific SpoIIQ expression using *PspoIIQ*-mCherry) in the WT, Δ*sda*, and Δ*kinC* strains at single-cell levels. We used a fluorescent reporter encoding a proteolytically unstable GFP-LCN to monitor *tapA* expression by minimizing the parameter of GFP stability (with *PtapA-gfp-LCN*) (24, 42). To determine which cells are sporulating, we counted cells expressing forespore-specific mCherry driven by the *spoIIQ* promoter since *spoIIQ* is expressed only during sporulation in the forespore (43) (see Supplemental Methods for details). Fluorescent images of the cells cultured in MSgg were taken at 8 h (Fig. 6B). Then, using a GFP threshold, we calculated the fraction of *tapA*-expressing cells in sporulating and nonsporulating cells for each strain (Fig. 6C). Among all cells, the overall fraction of *tapA*-expressing cells in the WT strain (0.23) was lower than that in the Δ*sda* strain (0.43) but higher than that in the Δ*kinC* strain (0.03) (Fig. 6C). Moreover, in all of these strains, this fraction of *tapA*-expressing cells was higher in nonsporulating cells than in sporulating cells (Fig. 6C); the difference in the fraction between sporulating and nonsporulating cells is statistically significant for WT (*P* = 4e-5) and for Δ*kinC* (*P* = 0.03) but not for Δ*sda* (*P* = 0.14). Furthermore, when we compared the sporulating (spo) and nonsporulating (non-spo) cells, the fold change in the fractions of *tapA*-expressing cells was smaller in the Δ*sda* (non-spo/spo, 0.42/0.41 = 1.01) than in the Δ*kinC* (non-spo/spo, 0.04/0.03 = 1.3) and WT (non-spo/spo, 0.19/0.09 = 2.1) strains. In comparison to the WT, weakening of mutual exclusiveness is statistically significant for Δ*sda* (fold change in fraction of *tapA*-expressing cells between non-spo and spo cells is larger in WT than in Δ*sda*, *P* = 1e-7) but not statistically significant in Δ*kinC* (*P* = 0.09). These experimental results were qualitatively consistent with the model predictions (Fig. 6A).

To summarize, our results provide another way to explain the apparent mutually exclusive cell fates between biofilm matrix production and sporulation. At later stages of starvation, a slowdown of growth rate leads to an increase in sporulation probability. Meanwhile, the probability of *tapA* expression would decrease due to the effect of slow growth on the SinR/SlrR ratio, leading to increased SinR. As a result, the probability that a sporulating cell also activates *tapA* expression is low, so the sporulation and matrix production appear as mutually exclusive cell fates. In the Δ*sda* strain, due to higher Spo0A~P levels with increased KinA activity, the fraction of the *tapA*-expressing cells is higher than that in the WT strain (Fig. 6). Moreover, in the Δ*sda* strain, the threshold of growth rate for sporulation is lower, and thus, the sporulation happens earlier than in the WT strain. As a result, the increase of the fraction of *tapA*-expressing cells in the Δ*sda* strain is more significant in sporulating cells than in nonsporulating cells. As our previous work shows (11), in the Δ*kinC* strain, Spo0A~P levels are lower than in the WT strain at early times of starvation, so the fraction of the *tapA*-expressing cells is lower than that in the WT strain (Fig. 6). Moreover, KinC acts as a sink of phosphoryl groups in the slow-growing cells in a culture population (11). As a result, sporulation happens earlier in the Δ*kinC* strain with increased levels of Spo0A~P at relatively early times of starvation. The acceleration of sporulation in the Δ*kinC* strain, therefore, is similar in the mechanism but weaker than that in the Δ*sda* strain. As a result, the deletion of *kinC* (Δ*kinC*) only slightly “weakens” the mutual exclusiveness of matrix production and sporulation.

## DISCUSSION

In a community of B. subtilis cells, at the onset of starvation, a subset of cells activates matrix production, leading to biofilm formation. However, at later stages of starvation, cells stop producing matrix and initiate sporulation. The master regulator Spo0A and the SinI-SinR-SlrR network play a critical role in the regulation of biofilm matrix production (18, 25, 44). In this work, we showed that the cellular growth rate would affect matrix production via an incoherent feed-forward loop (Fig. 3D). On the one hand, the slowdown of growth rate activates matrix production via an increase of Spo0A~P level and induction of SinI. On the other hand, the slowdown of growth represses matrix production by affecting the dosage between SinR and SlrR.

At the early stages of starvation, the slowdown of growth rate leads to the increase of Spo0A~P concentration via its effect on KinA concentration and the DNA replication cycle (14, 32). Our previous work shows that the noise of growth rate would affect the distribution of Spo0A~P in a culture population and thereby affects the heterogeneity of *tapA*-expressing cells in a culture population (11). Here, our model predicts that Spo0A~P is necessary for the expression of matrix genes, but it is not sufficient for the activation of matrix production (Fig. 2C). When Spo0A~P is low, the SinI-SinR-SlrR network only has a high-SinR-low-SlrR steady state, and *tapA* expression cannot be activated due to repression by SinR. When Spo0A~P increases, the SinI-SinR-SlrR network enters the bistable region (Fig. 2C), so the *tapA* expression could be activated by decreasing the SinR level via the stochastic fluctuations in the SinI-SinR-SlrR network. The bistability of the SinI-SinR-SlrR network ensures that only a subset of cells can activate matrix production, reducing the cost of matrix production and saving resources as a division of labor strategy (45, 46). The fluctuations in the SinI-SinR-SlrR network were known to be critical for determining the transition between the matrix-producing state and non-matrix-producing states (25, 30). Our model qualitatively reproduces the distribution of *tapA* expression in individual cells (Fig. 1D; Fig. S1), indicating that the heterogeneity of matrix production in a starving community could be explained mostly by the noise in the growth rate and the fluctuations in the SinI-SinR-SlrR network.

At late stages of growth, activation of SinI expression by the Spo0A~P level would be saturated (27). The slowdown of growth rate at late stages affects matrix production mainly through the cellular concentration of SinR and SlrR (Fig. 3). Due to different gene locations of *sinR* and *slrR* and different protein stability of their gene products (Fig. 3A), changes in growth rate cause a DNA-replication-associated gene dosage effect (Fig. S2B and C). As a result, when the growth rate slows down, the concentration of SinR increases faster than that of SlrR, and eventually, the free and active form of SinR represses *tapA* expression at late times (Fig. 3C and D). Similar effects of growth rate can be found in other bacterial models of cellular regulatory networks (47–50).

Previously, it has been demonstrated that cells entering sporulation stop *tapA* expression (24). To explain this mutual relationship between sporulation and matrix production, the following two mechanisms have been proposed: first, high Spo0A~P levels negatively affect SinI expression and eventually repress matrix production, and second, the change in the gene dosage between SinR and SlrR during sporulation also represses matrix production (24). Following this explanation, we would expect that the *tapA* expression would start to decrease earlier in the Δ*sda* strain because the Spo0A~P level increases more rapidly and the sporulation starts earlier than in the WT strain (Fig. S3). On the other hand, in the Δ*kinA* strain, the Spo0A~P level is too low to trigger sporulation; thus, we would expect that the *tapA* expression would start to decrease at later times in the Δ*kinA* strain than that in the WT strain (Fig. S3). As Fig. 4 shows, however, our data show that the *tapA* expression would start to decrease at about the same time in the WT, Δ*kinA*, and Δ*sda* strains. Moreover, we showed that perturbing growth dynamics can change the time when *tapA* starts to decrease (Fig. 5). Our model also successfully explains conditions under which biofilm matrix production and sporulation appear mutually exclusive, which is consistent with experimental results (Fig. 6). The model suggests that, rather than sporulation and asymmetric division as proposed in the previous study (24), the slowdown of growth rate is a major control mechanism to change the dosage of SinR and SlrR, eventually leading to repression of *tapA* expression.

In summary, our results provide a system-level understanding of the role of growth rate in controlling biofilm matrix production. By controlling the Spo0A~P level and the dosage of SinR and SlrR, the slowdown of growth rate regulates biofilm matrix production via an incoherent-feed-forward network. This proposed model explains the population-level dynamics and single-cell-level heterogeneity of matrix gene expression. Specifically, our study reveals that mutually exclusive cell fates between biofilm matrix production and sporulation can be generated by incoherent feed-forward regulatory networks. This network motif defines a signal-window (or a time-window for a time-dependent signal) during which matrix production is possible. Therefore, other cell fates that are activated by the same signal but with a threshold outside this window, e.g., sporulation, will occur only in the cells that are not expressing matrix. In other words, the cell fates appear mutually exclusive without explicitly inhibiting one another. Such a mechanism can be advantageous for cell survival under unforeseen conditions as a bet-hedging (51) or division of labor (45, 46) strategy and could be applicable in a wide range of other biological systems.

## MATERIALS AND METHODS

### Computational modeling methods.

**(i) Modeling the effect of growth rate on the gene copy number.** Under our experimental conditions, the growth rate is relatively slow, so the multifork replication is not considered. For simplicity, we assume that the replication starts right after cell division and the replication has a constant speed. Thus, the time before the replication of a certain gene is given by *t* = *p*τ_c_, where *p* represents the position of the gene, i.e., the normalized distance between the gene and the replication origin (*P *= 0 corresponds to the replication origin, and *P* = 1 corresponds to the replication terminus), and τ_c_ is the length of the C period. In the stochastic model, this time is explicitly included; the gene copy number is doubled at this specific time (Fig. S2B and C). In the deterministic model, following our previous work (11), we used an approximated average copy number:

$$n=2^{\left(1-\tau_{c}/\tau_{\mathrm{cyc}}\right)}$$

where $\tau_{\mathrm{cyc}}=\ln\left(2\right)/\mu$ is the length of the cell cycle. Following our previous model (15), we assume that τ_c_ is phenomenologically related to the growth rate *μ* as *τ_c_* = 0.78[h] + 0.15*/μ* (Fig. S2A). The positions of the genes involved in this work were shown in Table S2 in the supplemental material based on the data taken from the Biocyc database (52).

10.1128/msystems.00622-22.9 TABLE S2 Reactions and parameters of the stochastic model of the SinI-SinR-SlrR network. Download Table S2, PDF file, 0.1 MB. Copyright © 2023 Chen et al. 2023 Chen et al. https://creativecommons.org/licenses/by/4.0/ This content is distributed under the terms of the Creative Commons Attribution 4.0 International license.

**(ii) Modeling the growth dynamics and heterogeneity.** Following our previous work (2), we used a Moser-type model (8) to describe the growth dynamics:

(1) $$\frac{\mathrm{dC}}{\mathrm{dt}}=C\left(a\frac{k_{g}N^{h1}}{N^{h1}+K_{1}^{h1}}-\frac{k_{d}N^{h2}}{N^{h2}+K_{1}^{h2}}\right)$$

(2) $$\frac{\mathrm{dN}}{\mathrm{dt}}=-\gamma C\left(\frac{k_{g}N^{h1}}{N^{h1}+K_{1}^{h1}}-\psi \frac{k_{d}N^{h2}}{N^{h2}+K_{1}^{h2}}\right)$$

Here, *C* denotes the cell density in the units of OD and *N* denotes nutrient density in arbitrary units. The parameters include *k_g_* and *k_d_*, which are the maximum growth rate and death/sporulation rate, respectively; *K_1_* (*K_2_*) and *h_1_* (*h_2_*) are the half-saturation concentrations and Hill coefficients of cell growth and death (sporulation); γ is the yield coefficient; ψ is the fraction of the nutrient released by cell death/sporulation. The initial value of *N* was normalized to 1 and then *K_1_* (*K_2_*) was fitted. To be consistent with the experiment, *C(0)* = 0.1. The parameters used for the original growth dynamics and alternative growth dynamics (Fig. 4A) parameters were shown in Table S1 in the supplemental material.

10.1128/msystems.00622-22.8 TABLE S1 Parameters of the model for the growth dynamics. Download Table S1, PDF file, 0.1 MB. Copyright © 2023 Chen et al. 2023 Chen et al. https://creativecommons.org/licenses/by/4.0/ This content is distributed under the terms of the Creative Commons Attribution 4.0 International license.

Following our previous work (11), we assume that the distribution of generation times (*τ_cyc_*) of B. subtilis cells could be approximated by a normal distribution with CV = 0.25. The generation times of daughter cells were sampled from this distribution at the time of division with. The mean generation time is calculated based on the mean growth rate determined via the growth dynamics model, i.e.,

(3) $$\mu (t)=\frac{k_{g}N{\left(t\right)}^{h1}}{N{\left(t\right)}^{h1}+K_{1}^{h1}}$$

To avoid unrealistically high growth rates, we discarded the generation times below 0.2 h.

**(iii) The model of the SinI-SinR-SlrR network.** The model of the SinI-SinR-SlrR network contains the transcription, translation, and posttranslation reactions of SinI, SinR, and SlrR. SinR binds to specific DNA sequences as a tetramer (21). Following reference 53, we assumed that SinR is in the equilibrium between the tetramer and dimer. SinI is in the equilibrium between the monomer and dimer, and SinI dimer could bind to the SinR dimer and form a heterodimer (21, 53). The molecular basis of the binding between SlrR and SinR is not clear. For simplicity, following reference 21, we assumed that SlrR is predominately dimeric and SlrR dimer binds to SinR dimer forming a heterotetramer.

The kinetic parameters of the protein-protein interactions between SinI, SinR, and SlrR were estimated from *in vitro* experiments (23, 53). Moreover, SlrR is known to be quickly degraded *in vivo*; the degradation rate of SlrR was set to 0.6 h^−1^ (35). The degradation rate of other stable proteins was set to 0.2 h^−1^ (54) The transcription and translation of SinI, SinR, SlrR, and TapA were explicitly included. The relative transcription rates were chosen to ensure the bifurcation diagram resulted in the transitions from monostable to bistable regime come at the realistic growth rate and Spo0A~P level (Fig. 2). The absolute values of transcription rates for each mRNA were set to get the appropriate level of noise in stochastic simulations to enable stochastic activation and deactivation of matrix production in the bistable regime. Most half-lives of mRNA in B. subtilis are about 3 to 7 min (55). In this model, the degradation rates of all mRNAs were set to 8.3 h^−1^ (5-min half-life). Following reference 48, we assumed that the translation rate is independent of the growth rate. For simplicity, the translation rates of all the proteins were set to 200 h^−1^, similar to reference 56. The transcription rate of the TapA-GFP reporter was estimated by comparing the gfp-reporter fluorescent intensity between PtapA-gfp and kinA-gfp fusion (28) and the gfp-reporter fluorescent intensity. The reactions and parameters used in this model are listed in Table S2.

**(iv) Deterministic simulation of the model of the SinI-SinR-SlrR network.** To get the steady-state concentration of the species in the SinI-SinR-SlrR network (Fig. 1B, Fig. 3C), we solved the system numerically. The bistability of the system is determined by the number of the solutions for steady-states in numerical integration. The bifurcation graphs (Fig. 3B, Fig. 4A) were generated by numerically integrating the system with parameter continuation of growth rate and Spo0A~P level.

To qualitatively predict the dynamics of the tapA expression level using a traditional model (Fig. S4), we assume that high Spo0A~P concentration represses tapA expression with a Hill function:

$$P_{\mathrm{tapA}}\propto \frac{{\left[\text{Spo}0\text{A}\sim \text{P}\right]}^{n_{t1}}}{{\left[\text{Spo}0\text{A}\sim \text{P}\right]}^{n_{t1}}+K_{t1}^{n_{t1}}}\frac{K_{t2}^{n_{t2}}}{{\left[\text{Spo}0\text{A}\sim \text{P}\right]}^{n_{t2}}+K_{t2}^{n_{t2}}}$$

Here, we set n_t1_ = 1.5, n_t2_ = 8, K_t1_ = 0.15, and K_t2_ = 0.8.

**(v) Stochastic simulation of the model of SinI-SinR-SlrR network.** We modified a Python module stochpy (57) to simulate the model described in section “The model of the SinI-SinR-SlrR network” using the next-reaction algorithm (58). The parameters for stochastic simulation (Table S2) were converted from the parameters for a deterministic model assuming the cell volume is 4 fL (28).

To capture the decrease of growth rate during starvation, we simulated cell cycles separately. At the beginning of each cell cycle, the duration of the cell cycle, τ_cyc_, was sampled from a normal distribution determined by the current simulation time t. For simplicity, we assumed that the noise in the growth rate is independent of the noise in the SinI-SinR-SlrR system and the growth rate remains constant during the same cell cycle. Once the duration of the cell cycle *τ_cyc_* is sampled, the Spo0A~P level was then calculated for different strains based on the growth rate [μ = ln (2)/*τ_cyc_*] using the deterministic model described in our previous work (Fig. 2B) (2). The stochastic simulation was then performed from *t* to *t* + *τ_cyc_*. At the end of each cell cycle, the cell volume was partitioned evenly, and the species were binomially distributed between both daughter cells. Then one of the daughter cells was selected for the simulation of the next cell cycle, and the simulation time was updated as *t *=* t* + *τ_cyc_*. Then the simulation was performed iteratively on the new cell cycle until the simulation time reached a certain threshold. The iterative simulation process was illustrated in Fig. S6A in the supplemental material.

10.1128/msystems.00622-22.6 FIG S6 Stochastic simulation of the model. (A) The algorithm of the stochastic simulation for the starving condition. (B) The correction of the growth rate is used in the stochastic simulation. The red line shows the growth rate given by the deterministic model, which is the same as that in Fig. 2A. The dashed line shows the population growth rate reproduced by the stochastic model without correction. The blue line shows the population growth rate reproduced by the stochastic model with *ϵ *= 0.2. Download FIG S6, PDF file, 0.7 MB. Copyright © 2023 Chen et al. 2023 Chen et al. https://creativecommons.org/licenses/by/4.0/ This content is distributed under the terms of the Creative Commons Attribution 4.0 International license.

To accurately simulate the effect of growth rate on the gene dosage, gene replication was explicitly included in the model. The replication times of genes were calculated with the model described above in section “(i) Modeling the effect of growth rate on the gene copy number” at such times, the copy number of genes was doubled. At the beginning of the new cell cycle, the copy number of each gene was reset to 1.

The population growth rate was assumed to be a function of the simulation time. According to Equation 1, we set time-dependent growth rate as in Equation 3. We assumed that the duration of cell cycles exists at time *t* and follows a normal distribution with the mean = ln(2)/μ(t) and the coefficient of variation (std/mean), CV = 0.25. Our simulation determines τ_cyc_ at the beginning of the cell cycle. Thus, the cell cycles existing at time *t* would start prior to *t.* As the result, the mean duration of cell cycles starting at time *t*_s_ should correspond to the mean duration of cell cycles existing at a later time *t > t_s_*. To correct for this offset, we phenomenologically introduced a time-shift term in the τ_cyc_ calculation as follows:

$$\tau_{\mathrm{cyc}}(t)=\frac{\ln\left(2\right)}{\mu \left(t+\delta t\right)};\;\delta t=\frac{\epsilon \ln\left(2\right)}{\mu (t)}$$

We run the stochastic simulation of the cell cycle duration for 500 cell lineages to reproduce the dynamics population growth rate. The population growth rate at time *t* is given by the average growth rate of all the cell cycles existing at time *t*. As Fig. S6B shows, ϵ is set to 0.2 to get a good phenomenological approximation.

**(vi) Single-cell sporulation and matrix expression.** According to reference 15, the sporulation probability of B. subtilis cells is highly correlated with growth rate and Spo0A~P level. To predict the fraction of *tapA*-expressing cells in sporulating and nonsporulating cells (Fig. 6A), we consider the cells with growth rates lower than a threshold (0.17 h^−1^) to be “sporulating cells” (15). The cells with a TapA amount in excess of 500 molecules (corresponds to the first bin in Fig. 2D) were considered “tapA-expressing cells.” All the cells at T8 are labeled as spo/non-spo and TapA on/off and were counted (Fig. 6A).

To get the distribution of TapA levels at different times (Fig. 2D), for each strain, we performed 1,000 runs of stochastic simulation for each strain. To test the behavior of the system under certain conditions (Fig. S3), the model is simulated with a fixed Spo0A~P level and growth rate. For each condition, the model is simulated for a long time (>200 h), and SinR and SlrR levels are sampled at an arithmetic sequence of time points with a common difference of 0.5 h.

**(vii) Simulations used to produce computational figures.**

- Fig. S2 was generated by modeling the effect of growth rate on the gene copy number (section “Modeling the effect of growth rate on the gene copy number”).
- Fig. 2A and 5A were generated by modeling the growth dynamics and heterogeneity (section “Modeling the growth dynamics and heterogeneity”).
- Fig. 2B and C and 4A (lines) and Fig. S4B were generated by the deterministic model of phosphorelay (11). The reactions and parameters in this model can be found in Table S1 of reference 11. To simulate the gene deletion mutant, the production rate of the corresponding protein was set to zero. To simulate the dynamics as a function of time, results of Fig. 2A were employed.
- Fig. 1B; 3A, B, and C; and 4A (shading) and Fig. S4C were generated by solving the deterministic model of the SinI-SinR-SlrR network (section “Deterministic simulation of the model of the SinI-SinR-SlrR network”) for fixed values of Spo0A~P and/or for growth rate as indicated. To emphasize the effect of growth rate on the SinR/SlrR ratio (Fig. 3A), the repression of SlrR expression by SinR was not modeled.
- Fig. 1C and Fig. S3 were generated by stochastic simulation of the SinI-SinR-SlrR network (Table S2) for a fixed value of Spo0A~P as indicated.
- Fig. 2D, 4B, 5B, and 6A and Fig. S5 and S7 were generated by stochastic simulation of the SinI-SinR-SlrR network with stochastic and time-varying growth rates as described in section “Single-cell sporulation and matrix expression.” The cell fates for Fig. 6A are determined as described in section “Simulations used to produce computational figures.”

### Strains, plasmids, and oligonucleotide DNAs.

The strains, plasmids, and oligonucleotide DNAs used are listed in Table S3 in the supplemental material. B. subtilis strains used in this work are isogenic derivatives of the undomesticated and competent DK1042 (59). DK1042 is a derivative of strain NCIB 3610 forming a biofilm matrix (60). All mutant strains of B. subtilis were constructed by transformation with either chromosomal DNA or plasmid DNA as described by Harwood and Cutting (61). The standard recombinant DNA techniques, including plasmid DNA construction and isolation using Escherichia coli DH5α were performed as described by Sambrook and Russell (62). Plasmid pMF523 (*amyE::PspoIIQ-mCherry spc*) was constructed by ligating the PCR fragment containing the *spoIIQ* promoter and the coding region of mCherry into pDG1730 (63). The *spoIIQ* promoter region was amplified by PCR with primers omf42 and omf43 using chromosomal DNA from B. subtilis PY79 as the template. The mCherry coding region was amplified by PCR with primers om87 and om88 using pDR201 (64) as the template. The two PCR products were recovered from the agarose gel and purified using the gel extraction kit (Qiagen). The purified *PspoIIQ* DNA fragment was digested with EcoRI and HindIII. The purified mCherry DNA fragment was digested with HindIII and BamHI. The digested DNA fragments were purified by the PCR purification kit (Qiagen). The purified *spoIIQ* promoter and mCherry DNA fragments were mixed and ligated with pDG1730 digested with EcoRI and BamHI. The resulting plasmid was integrated into the *amyE* locus of the B. subtilis chromosomal DNA by double-crossover homologous recombination. Plasmid pMF1154 (*thrC::PtapA-gfp-lcn erm*) was constructed by ligating the PCR fragment containing the *tapA* promoter and the coding region of GFP-LCN into pDG1664 (63). The *tapA* promoter region was prepared from pMF712 with EcoRI and HindIII digestion (12). The digested DNA fragment containing the *tapA* promoter was recovered from the agarose gel and purified using the gel extraction kit (Qiagen). The GFP coding region was amplified by PCR with primers omf316 and om528 using pMF719 (*thrC::PtapA-gfp erm*) as the template (11). The PCR product containing *gfp-lcn* was recovered from the agarose gel and purified using the gel extraction kit (Qiagen). The purified PCR product was digested with HindIII and BamHI and purified by the PCR purification kit (Qiagen). The purified *tapA* promoter and *gfp-lcn* DNA fragments were mixed and ligated with pDG1664 digested with EcoRI and BamHI. The resulting plasmid was integrated into the *thrC* locus of the B. subtilis chromosomal DNA by double-crossover homologous recombination. Plasmid pMF713 (*amyE::PepsA-lacZ spc*) was constructed by ligating the PCR fragment containing the epsA promoter and pDG1728 (63). The *epsA* promoter region was amplified by PCR with primers om210 and om211 using chromosomal DNA from B. subtilis PY79 as the template. The PCR product was recovered from the agarose gel and purified using the gel extraction kit (Qiagen). The purified DNA fragment was digested with EcoRI and HindIII. The digested DNA fragment was purified by the PCR purification kit (Qiagen). The purified DNA fragment was ligated with pDG1728 digested with EcoRI and HindIII.

10.1128/msystems.00622-22.10 TABLE S3 List of strains, plasmids, and oligonucleotides used in this work. Download Table S3, PDF file, 0.2 MB. Copyright © 2023 Chen et al. 2023 Chen et al. https://creativecommons.org/licenses/by/4.0/ This content is distributed under the terms of the Creative Commons Attribution 4.0 International license.

### Media and culture conditions.

Lysogeny broth (LB) medium (62) was used for routine growth of E. coli and B. subtilis. Minimal salts glycerol glutamate (MSgg) medium was used for biofilm matrix production and sporulation of B. subtilis (2). For nitrogen-depleted MSgg medium, l-glutamate was 10-fold diluted (0.05% final concentration, relative to the original 0.5% final concentrations). Cells were cultured with shaking (150 rpm) overnight in LB (5 mL) at 28°C. The overnight culture was transferred to fresh LB (10 mL) to an optical density at 600 nm (OD_600_) of 0.05. The fresh culture was incubated at 37°C with shaking (150 rpm) to the mid-log phase (OD_600_ ≈ 0.5) to synchronize cell growth. Then, the fresh culture was transferred to MSgg (20 mL) to an OD_600_ of 0.05 and incubated in a culture flask at 37°C with shaking (150 rpm). Culture samples were collected at the indicated time points and assayed for a specific activity of a β-galactosidase reporter or processed for microscopy. Cell growth in liquid media was measured using a spectrophotometer by reading the OD_600_. Strains harboring reporter genes at the nonessential *thrC* locus were supplemented with 1 mg mL^−1^ of l-threonine in the MSgg medium. When making solid agar medium, 1.5% (wt/vol) agar was included. Antibiotics were used for the selection of transformants at the following concentrations: 10 μg mL^−1^ of tetracycline, 100 μg mL^−1^ of spectinomycin, 20 μg mL^−1^ of kanamycin, 5 μg mL^−1^ of chloramphenicol, and 1 μg mL^−1^ of erythromycin.

### β-Galactosidase assay.

B. subtilis undomesticated strains were grown in a liquid medium as described in the above section “Media and culture conditions.” Samples were collected at indicated time points, and β-galactosidase assays were performed as described previously (11). The mean activities of at least three independent experiments are shown with standard deviations.

### Microscopy analysis.

Cells collected at the specified times were spotted onto MSgg medium containing 1% (wt/vol) agarose (ISC Bioexpress; E-3119-500) in a Gene Frame chamber (Thermo Scientific; AB-0577; 65 mL, 1.5 by 1.6 cm) and covered by a cover glass. The cell samples were examined immediately using a fluorescence microscope (Olympus; model BX61) with an Olympus UPlanFL N 100× microscope objective. GFP and mCherry fluorescence were visualized using Chroma 41017 and Olympus U-MWG2 filter sets, respectively. Typical exposure times were 200 ms. The microscope system was operated using SlideBook image analysis software (Intelligent Imaging Innovations, Inc.), and the resulting images were processed as described previously (11). Representative images are shown. GFP and mCherry channels are shown in green and magenta pseudocolors, respectively. Using the same method as those in reference 11, we segmented the cells and calculated the pixel-wise mean fluorescence intensity of GFP and mCherry for each cell. For each image, the pixel-wise mean GFP/mCherry intensity of the no-cell area was calculated as background. The cells with *PtapA* activity significantly higher than the background were considered “*tapA*-expressing cells,” and the cells with *PspoIIQ* activity significantly higher than the background were considered “sporulating cells.” For each strain, 3 parallel experiments for each strain were performed and 3 images containing between 600 and 1,600 cells in the field of view were analyzed to determine the fraction of cells with each reporter activated. The fractions of *tapA*-expressing cells were calculated with standard deviation of measurements from 9 images shown as error bars on Fig. 6. The fold change for the *tapA* fraction between sporulating (spo) and nonsporulating (non-spo) cells were calculated for each image. Then a two-sample *t* test was performed to compare data from pairs of strains (*n* = 9 data points for each group), and the resulting *P* values are listed in Results.

### Data availability.

Parameters used for simulations are included in Table S1 and S2. The code and data used in this work can be found online at https://doi.org/10.5281/zenodo.7544671.
