# Supplementary material for: The Slowdown of Growth Rate Controls the Single-Cell Distribution of Biofilm Matrix Production via an SinI-SinR-SlrR Network
Source: mSystems. 2023 Feb 14;8(2):e00622-22. doi: 10.1128/msystems.00622-22 (PMC10134886; doi:10.1128/msystems.00622-22)
Supplement: FIG S1 [file msystems.00622-22-s0001.pdf]

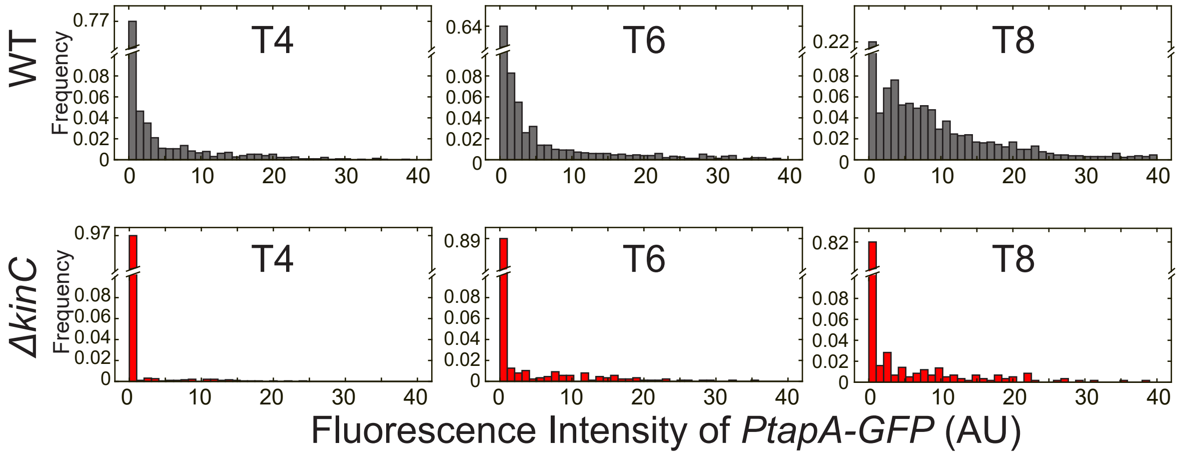

Figure S1: The experimentally observed distribution of *tapA* expression
The distribution of mean-fluorescence intensity of *PtapA-GFP* in WT and Δ*kinC* cells at T4, T6, and T8. The value of the first bin was labeled on each histogram. Reproduced using data from from [11].
