# Supplementary material for: The Slowdown of Growth Rate Controls the Single-Cell Distribution of Biofilm Matrix Production via an SinI-SinR-SlrR Network
Source: mSystems. 2023 Feb 14;8(2):e00622-22. doi: 10.1128/msystems.00622-22 (PMC10134886; doi:10.1128/msystems.00622-22)
Supplement: FIG S5 [file msystems.00622-22-s0005.pdf]

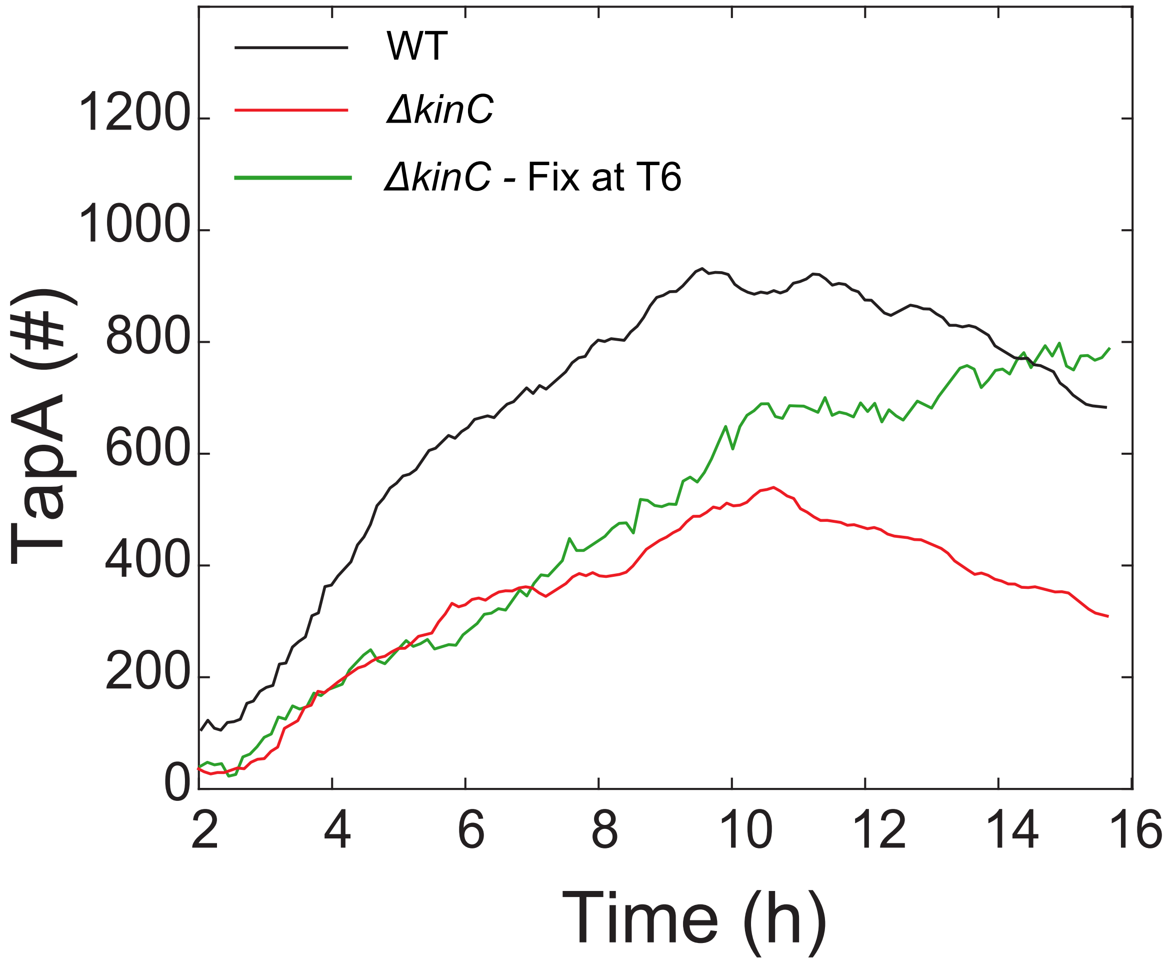

Figure S5: *tapA* expression is not in a steady-state during simulation. The red and black line shows the dynamics of mean TapA levels of WT and $\Delta kinC$ strains, which are same as Fig. 4B. The green line shows the mean TapA levels of $\Delta kinC$ strain supposing the growth rate stops to decrease and fixed at T6.
