# Supplementary material for: The Slowdown of Growth Rate Controls the Single-Cell Distribution of Biofilm Matrix Production via an SinI-SinR-SlrR Network
Source: mSystems. 2023 Feb 14;8(2):e00622-22. doi: 10.1128/msystems.00622-22 (PMC10134886; doi:10.1128/msystems.00622-22)
Supplement: FIG S2 [file msystems.00622-22-s0002.pdf]

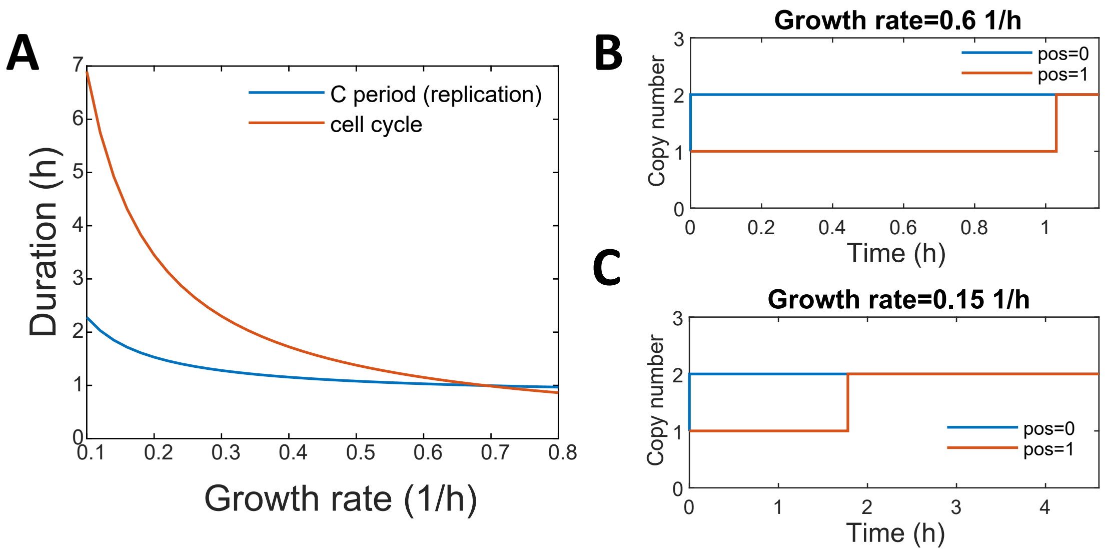

Figure S2: Growth rate affects gene dosage for the replication-terminus proximal genes. (A) The duration of the C period (replication) and the whole cell cycle as functions of growth rate. (B and C) The change of the copy numbers of a replication-terminus proximal gene ($p = 1$) and a replication-origin proximal gene ($p = 0$) within a cell cycle. The copy numbers were plotted for growth rates equal to 0.6 h^{-1} (B) and 0.15 h^{-1} (C). For a gene proximal to the replication origin, the gene dosage is 2 during the whole cell cycle regardless of the growth rate. For a gene proximal to the replication terminus, the fraction of time that the gene dosage is equal to 2 is larger when the growth rate is lower.
