# Supplementary material for: The Slowdown of Growth Rate Controls the Single-Cell Distribution of Biofilm Matrix Production via an SinI-SinR-SlrR Network
Source: mSystems. 2023 Feb 14;8(2):e00622-22. doi: 10.1128/msystems.00622-22 (PMC10134886; doi:10.1128/msystems.00622-22)
Supplement: FIG S3 [file msystems.00622-22-s0003.pdf]

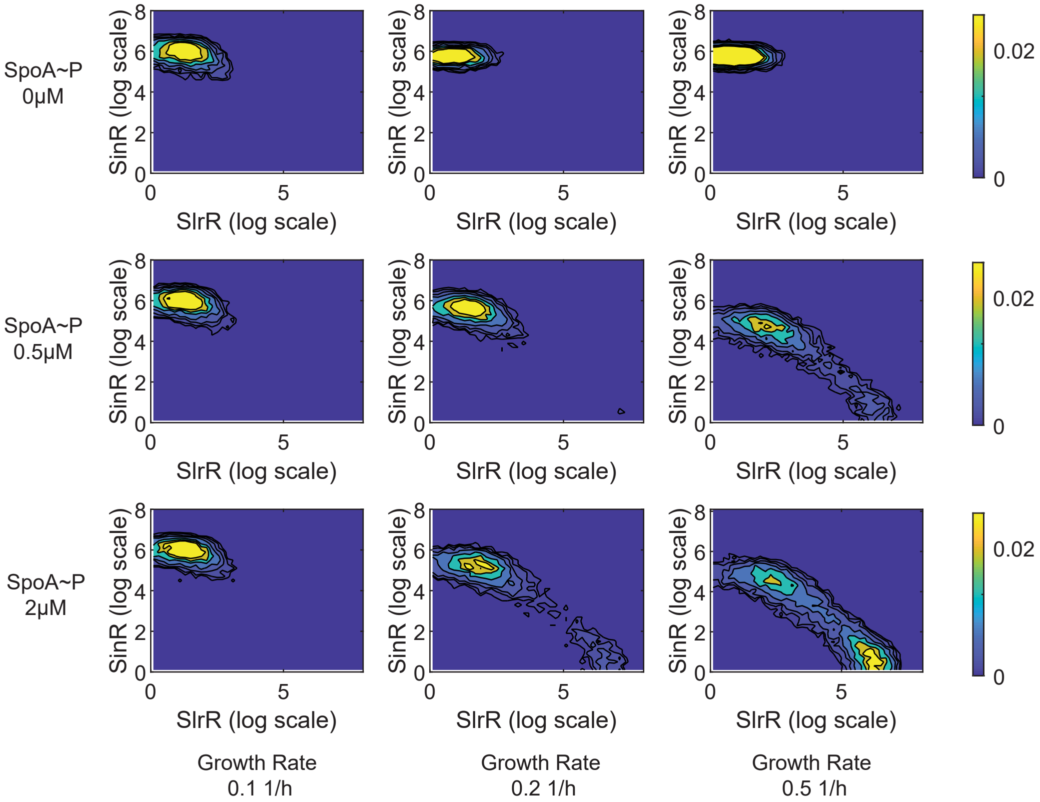

Figure S3: The distribution of SlrR and SinR levels at different Spo0A∼P levels and growth rates from stochastic simulations.
