# Supplementary material for: The Slowdown of Growth Rate Controls the Single-Cell Distribution of Biofilm Matrix Production via an SinI-SinR-SlrR Network
Source: mSystems. 2023 Feb 14;8(2):e00622-22. doi: 10.1128/msystems.00622-22 (PMC10134886; doi:10.1128/msystems.00622-22)
Supplement: FIG S4 [file msystems.00622-22-s0004.pdf]

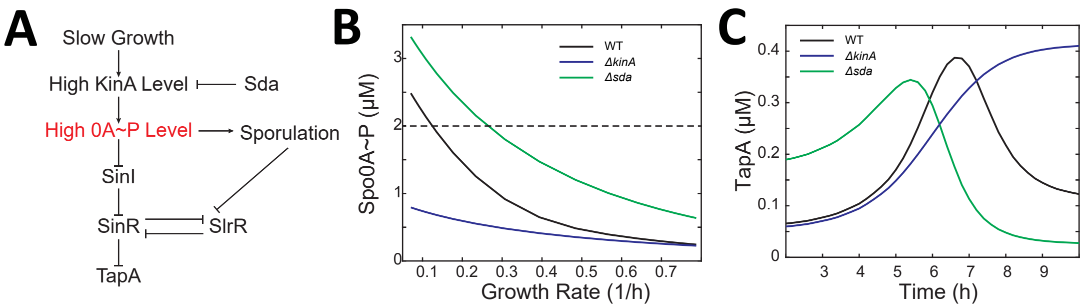

Figure S4: The traditional explanation of why *tapA* expression decreases at late times.
(A) The traditional model of how high Spo0A∼P levels repress *tapA* expression. On the one hand, high Spo0A∼P levels repress the expression of SinI. On the other hand, high Spo0A∼P levels trigger sporulation, which changes the dosage between SinR and SlrR and eventually represses the expression of *tapA*.
(B) The Spo0A∼P values as functions of growth rate in WT, Δ*kinA*, and Δ*sda* strains. The assumed threshold of Spo0A∼P level that is sufficient to represses *tapA* expression is shown as the dashed line.
(C) The predicted dynamics of *tapA* expression with the traditional model in WT, Δ*kinA*, and Δ*sda* strains.
