# Supplementary material for: The Slowdown of Growth Rate Controls the Single-Cell Distribution of Biofilm Matrix Production via an SinI-SinR-SlrR Network
Source: mSystems. 2023 Feb 14;8(2):e00622-22. doi: 10.1128/msystems.00622-22 (PMC10134886; doi:10.1128/msystems.00622-22)
Supplement: FIG S7 [file msystems.00622-22-s0007.pdf]

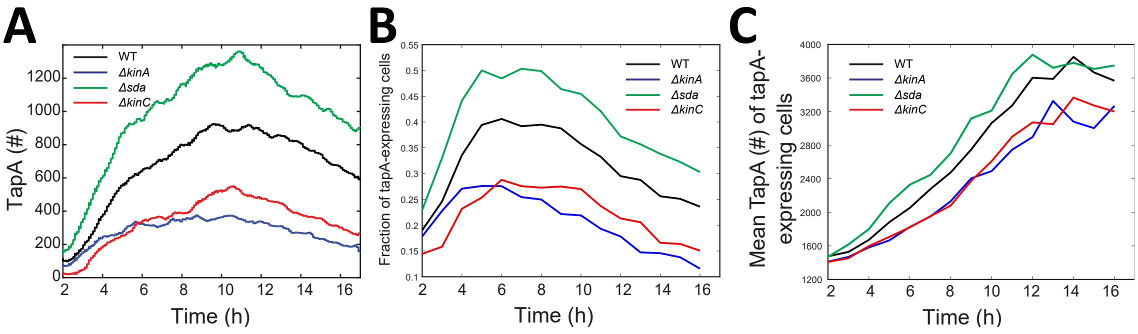

Figure S7: Effects of tapA-expressing cell fraction and *tapA*-expressing levels.

(A) Simulated *tapA* expression dynamics in different strains (Copied from Fig. 4B).

(B) Simulated dynamics of the fraction of *tapA*-expressing cells.

(C) Simulated dynamics of mean TapA level of all *tapA*-expressing cells.
