# Supplementary material for: The Slowdown of Growth Rate Controls the Single-Cell Distribution of Biofilm Matrix Production via an SinI-SinR-SlrR Network
Source: mSystems. 2023 Feb 14;8(2):e00622-22. doi: 10.1128/msystems.00622-22 (PMC10134886; doi:10.1128/msystems.00622-22)
Supplement: TABLE S2 [file msystems.00622-22-s0009.pdf]

Table S2 Reactions and parameters of the stochastic model of SinI-SinR-SlrR network.

Description	Reaction	Parameters	Reference
SinI dimerization	$2\text{SinI} \underset{k_{di}}{\overset{k_{bi}}{\rightleftharpoons}} \text{SinI}_2$	k_{bi}=0.032$\#^{-1}\text{h}^{-1}$* k_{di}=36h^{-1}	[7]
SinR tetramerization	$2\text{SinR}_2 \underset{k_{dr}}{\overset{k_{br}}{\rightleftharpoons}} \text{SinR}_4$	k_{bi}=0.032$\#^{-1}\text{h}^{-1}$ k_{di}=129 h^{-1}	[7]
SinI-SinR interaction	$\text{SinI}_2 + \text{SinR}_2 \xrightarrow{k_{ir}} 2\text{SinI} \cdot \text{SinR}$	k_{ir}=0.32 $\#^{-1}$ h^{-1}	[7]
SlrR-SinR interaction	$\text{SlrR}_2 + \text{SinR}_2 \xrightarrow{k_{lr}} \text{SlrR}_2 \cdot \text{SinR}_2$	k_{lr}=0.32 $\#^{-1}$ h^{-1}	[6]
DNA binding of SinR tetramer	$\text{Promoter} + \text{SinR}_4 \underset{k_{dpr}}{\overset{k_{pr}}{\rightleftharpoons}} \text{Promoter} \cdot \text{SinR}_4$	k_{pr}=0.6 $\#^{-1}$ h^{-1} k_{dpr}=5 h^{-1}	[2]
DNA binding of Spo0A∼P	$\text{Promoter} + \text{Spo0A}{\sim}\text{P} \underset{k_{dpa}}{\overset{k_{pa}}{\rightleftharpoons}} \text{Promoter} \cdot \text{Spo0A}{\sim}\text{P}$	k_{pa}=0.6 $\#^{-1}$ h^{-1} k_{dpa}=12.5 h^{-1}	[4]
Transcription of *sinI*	$PsinI \cdot \text{Spo0A}{\sim}\text{P} \xrightarrow{v_i} PsinI \cdot \text{Spo0A}{\sim}\text{P} + \text{mRNA}_{sinI}$	v_i=85 h^{-1}	**
Transcription of *sinR*	$PsinR \xrightarrow{v_r} PsinR + \text{mRNA}_{sinR}$	v_r=100 h^{-1}	**
Transcription of *slrR*	$PslrR \xrightarrow{v_l} PslrR + \text{mRNA}_{slrR}$	v_l=125 h^{-1}	**
Transcription of *tapA*	$PtapA \xrightarrow{v_t} PtapA + \text{mRNA}_{tapA}$	v_l=100 h^{-1}	[3]
Protein translation	$\text{mRNA} \xrightarrow{v_{tr}} \text{mRNA} + \text{Protein}$	v_{tr}=200 h^{-1}	**
mRNA degradation	$\text{mRNA} \xrightarrow{k_{dm}} \varnothing$	k_{dm}=8.3 h^{-1}	[5]
Protein degradation	$\text{Protein} \xrightarrow{k_{dp}} \varnothing$	k_{dm}=0.2 h^{-1}	[8]
SlrR degradation	$\text{SlrR}_2 \xrightarrow{k_{ds}} \varnothing$	k_{ds}=0.6 h^{-1}	[1]

* Here and below # denotes the number of molecules.

** Set as described in the Methods

References

[1] Y. Chai, R. Kolter, and R. Losick. Reversal of an Epigenetic Switch Governing Cell Chaining in Bacillus subtilis by Protein Instability. *Molecular microbiology*, 78(1):218–229, Oct. 2010.

[2] V. L. Colledge, M. J. Fogg, V. M. Levdikov, A. Leech, E. J. Dodson, and A. J. Wilkinson. Structure and Organisation of SinR, the Master Regulator of Biofilm Formation in Bacillus subtilis. *Journal of Molecular Biology*, 411(3-26):597–613, Aug. 2011.

[3] P. Eswaramoorthy, J. Dinh, D. Duan, O. A. Igoshin, and M. Fujita. Single-cell measurement of the levels and distributions of the phosphorelay components in a population of sporulating Bacillus subtilis cells. *Microbiology (Reading, England)*, 156(Pt 8):2294–2304, Aug. 2010.

[4] M. Fujita, J. E. González-Pastor, and R. Losick. High- and Low-Threshold Genes in the Spo0A Regulon of Bacillus subtilis. *Journal of Bacteriology*, 187(4):1357–1368, Feb. 2005.

[5] G. Hambraeus, C. von Wachenfeldt, and L. Hederstedt. Genome-wide survey of mrna half-lives in bacillus subtilis identifies extremely stable mrnas. *Molecular Genetics and Genomics*, 269(5):706–714, 2003.

[6] J. A. Newman, C. Rodrigues, and R. J. Lewis. Molecular basis of the activity of SinR protein, the master regulator of biofilm formation in Bacillus subtilis. *The Journal of Biological Chemistry*, 288(15):10766–10778, Apr. 2013.

[7] D. J. Scott, S. Leejeerajumnean, J. A. Brannigan, R. J. Lewis, A. J. Wilkinson, and J. G. Hoggett. Quaternary re-arrangement analysed by spectral enhancement: The interaction of a sporulation repressor with its antagonist. *Journal of Molecular Biology*, 293(5):997–1004, Nov. 1999.

[8] V. Sekar and J. H. Hageman. Protein turnover and proteolysis during sporulation of Bacillus subtilis. *Folia Microbiologica*, 32(6):465–480, 1987.
