# Supplementary material for: The Slowdown of Growth Rate Controls the Single-Cell Distribution of Biofilm Matrix Production via an SinI-SinR-SlrR Network
Source: mSystems. 2023 Feb 14;8(2):e00622-22. doi: 10.1128/msystems.00622-22 (PMC10134886; doi:10.1128/msystems.00622-22)
Supplement: TABLE S1 [file msystems.00622-22-s0008.pdf]

Table S1 Parameters of the model for the growth dynamics

parameter	original dynamics	alternative dynamics
k_g	1.90 h^{-1}	1.90 h^{-1}
k_d	0.622 h^{-1}	0.622 h^{-1}
ψ	0.3	0.3
h_1	1.69	1.69
h_2	2.99	2.99
K_1	1.21	0.46
K_2	0.132	0.132
γ	0.10	1.01

* In the model of growth dynamics, the cell density (which is represented by OD) and nutrient level are considered as dimensionless variables, so the parameters do not have units.
