# Supplementary material for: The Slowdown of Growth Rate Controls the Single-Cell Distribution of Biofilm Matrix Production via an SinI-SinR-SlrR Network
Source: mSystems. 2023 Feb 14;8(2):e00622-22. doi: 10.1128/msystems.00622-22 (PMC10134886; doi:10.1128/msystems.00622-22)
Supplement: FIG S6 [file msystems.00622-22-s0006.pdf]

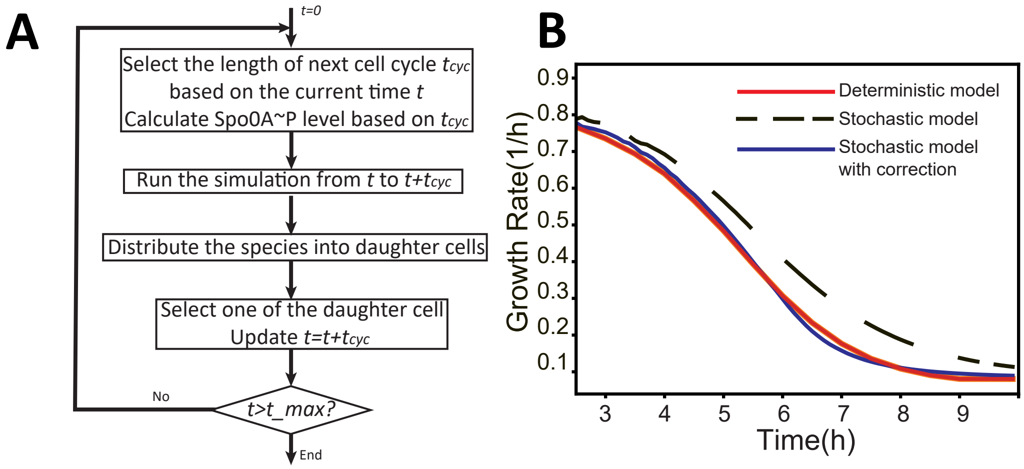

Figure S6: Stochastic simulation of the model.

(A) The algorithm of the stochastic simulation for the starving condition.

(B) The correction of growth rate used in the stochastic simulation. The red line shows the growth rate given by the deterministic model, same as Fig. 2A. The dashed line shows the population growth rate reproduced by the stochastic model without correction. The blue line shows the population growth rate reproduced by the stochastic model with $k = 0.2$.
