# Supplementary material for: The Slowdown of Growth Rate Controls the Single-Cell Distribution of Biofilm Matrix Production via an SinI-SinR-SlrR Network
Source: mSystems. 2023 Feb 14;8(2):e00622-22. doi: 10.1128/msystems.00622-22 (PMC10134886; doi:10.1128/msystems.00622-22)
Supplement: TABLE S3 [file msystems.00622-22-s0010.pdf]

Table S3 List of strains, plasmids, and oligonucleotides

***B. subtilis* strains used for β-galactosidase**			
**Strain**	**Genotype**	**Reference/Source**	**Construction***
**MF7463**	*amyE::PtapA-lacZ cm comI*^{Q12L}	This study	MF5762 → MF5609
**MF5764**	*amyE::PepsA-lacZ cm comI*^{Q12L}	This study	pMF713 → MF5609
**MF9089**	*Δsda::erm amyE::PtapA-lacZ cm comI*^{Q12L}	(Chen *et al.*, 2022)	MF6110 → MF7463
**MF9132**	*ΔkinA amyE::PtapA-lacZ cm comI*^{Q12L}	This study	MF5762 → MF8816
**MF9134**	*ΔkinC amyE::PtapA-lacZ cm comI*^{Q12L}	This study	MF5762 → MF8068

***B. subtilis* strains used for microscopy**			
**Strain**	**Genotype**	**Reference/Source**	**Construction**
**MF11412**	*thrC::PtapA-gfp-lcn erm amyE::PspoIIQ-mCherry spc comI*^{Q12L}	This study	MF3746 → MF11406
**MF11414**	*ΔkinC thrC::PtapA-gfp-lcn erm amyE::PspoIIQ-mCherry spc comI*^{Q12L}	This study	MF3746 → MF11408
**MF11415**	*Δsda thrC::PtapA-gfp-lcn erm amyE::PspoIIQ-mCherry spc comI*^{Q12L}	This study	MF3746 → MF11409

***B. subtilis* strains used for strain construction**			
**Strain**	**Genotype**	**Reference/Source**	**Construction**
**PY79**	Prototroph wild type Domesticated wild type strain	(Youngman *et al.*, 1984)	Laboratory stock
**MF5762**	*amyE::PtapA-lacZ cm*	(Chu *et al.*, 2006)	pDP280 → PY79
**MF5609**	*DK1042 comI*^{Q12L} (competent NCIB 3610) Undomesticated wild type	(Konkol *et al.*, 2013)	Daniel Kearns
**MF8816**	*ΔkinC comI*^{Q12L}	(Chen *et al.*, 2022)	Laboratory stock
**MF8068**	*ΔkinA comI*^{Q12L}	(Chen *et al.*, 2022)	Laboratory stock
**MF3746**	*amyE::PspoIIQ-mCherry spc*	This study	pMF523 → PY79
**MF11406**	*thrC::PtapA-gfp-lcn erm comI*^{Q12L}	This study	11404 → 5609
**MF11408**	*ΔkinC thrC::PtapA-gfp-lcn erm comI*^{Q12L}	This study	11404 → 8068
**MF11409**	*Δsda thrC::PtapA-gfp-lcn erm comI*^{Q12L}	This study	11404 → 10691
**MF11404**	*thrC::PtapA-gfp-lcn erm*	This study	pMF1154 → PY79
**MF10691**	*Δsda comI*^{Q12L}	This study	pDR244 → 9099
**MF9099**	*Δsda::erm comI*^{Q12L}	This study	6110 → 5609
**MF6110**	*BKE25690 Δsda::erm BSU25690*	BGSC[†]	BGSC

Plasmids used for strain construction		
**Plasmid**	**Genotype**	**Reference/Source**
**pDP280**	*amyE::PtapA-lacZ cm*	(Chu *et al.*, 2006)
**pMF713**	*amyE::PepsA-lacZ spc*	This study
**pMF523**	*amyE::PspoIIQ-mCherry spc*	This study
**pMF1154**	*thrC::PtapA-gfp-lcn erm*	This study
**pDR244**	encoding cre recombinase to remove the antibiotic resistance cassette	(Koo *et al.*, 2017)

Oligo nucleotides used for plasmid construction	
**Primer**	**Sequence**
omf42	5'-gcggaattccggtatcggctgttaccatt-3'
omf43	5'-cgcaagcttcagcaacattctgaacacttt-3'
om87	5′-gccaagcttacataaggaggaactact atggtcagcaagggagaggaagat-3′
om88	5′-gccggatccttatttgtataattcgtccattccacctgt-3′
om210	5′-cgcgaattccgccattcttcttcaccgccgctgattt-3′
om211	5′-cgcaagcttcggctggcttcccgcgcccctttctg-3′
omf316	5'-gccaagcttacataaggaggaactactatgagtaaaggagaagaactt-3'
om528	5′-gccggatccttagttgcacagtttgtatagttcatccatgccatg-3′

*Arrows indicate transformation and point from donor DNA to recipient strain.
†Bacillus Genetic Stock Center

References

Chen, Z., P. Srivastava, B. Zarazua-Osorio, A. Marathe, M. Fujita & O.A. Igoshin, (2022) *Bacillus subtilis* Histidine Kinase KinC Activates Biofilm Formation by Controlling Heterogeneity of Single-Cell Responses. *mBio*: e0169421.

Chu, F., D.B. Kearns, S.S. Branda, R. Kolter & R. Losick, (2006) Targets of the master regulator of biofilm formation in *Bacillus subtilis*. *Molecular microbiology* **59**: 1216-1228.

Konkol, M.A., K.M. Blair & D.B. Kearns, (2013) Plasmid-encoded ComI inhibits competence in the ancestral 3610 strain of *Bacillus subtilis*. *Journal of bacteriology* **195**: 4085-4093.

Koo, B.M., G. Kritikos, J.D. Farelli, H. Todor, K. Tong, H. Kimsey, I. Wapinski, M. Galardini, A. Cabal, J.M. Peters, A.B. Hachmann, D.Z. Rudner, K.N. Allen, A. Typas & C.A. Gross, (2017) Construction and Analysis of Two Genome-Scale Deletion Libraries for *Bacillus subtilis*. *Cell systems* **4**: 291-305 e297.

Youngman, P., J.B. Perkins & R. Losick, (1984) Construction of a cloning site near one end of Tn917 into which foreign DNA may be inserted without affecting transposition in *Bacillus subtilis* or expression of the transposon-borne erm gene. *Plasmid* **12**: 1-9.
